# Targeting HDACs with dacinostat amplifies radiation-induced DNA damage via sustained phosphorylated p53 and repair inhibition in aggressive meningioma

**DOI:** 10.1038/s41419-026-08899-4

**Published:** 2026-06-02

**Authors:** Shahana Shaji, Juri Na, C. Oliver Hanemann

**Affiliations:** Peninsula Medical School, Faculty of Health, Plymouth University, Devon, UK

**Keywords:** Radiotherapy, DNA damage response, Cellular neuroscience, Double-strand DNA breaks

## Abstract

The lack of standard systemic treatments in meningioma presents a therapeutic challenge, making radiotherapy a crucial intervention for recurrent, high-grade, or inoperable tumours. Nonetheless, the efficacy of radiotherapy is constrained by the inherent radioresistance of meningiomas, attributed to their proficient DNA damage repair and robust cell cycle checkpoint regulation. These biological barriers, along with anatomical constraints of avoiding damage to adjacent neural tissues, restrict dose escalation and therapeutic gains. Histone deacetylases (HDACs) are mediators of radioresistance in meningioma, and their inhibition can be a rational approach to enhance the therapeutic efficacy. This study systematically investigated the radiosensitising potential of dacinostat, a pan-HDACi, and demonstrated its superior synergy with radiation compared to other FDA-approved HDACis. Pre-treatment with dacinostat effectively suppressed radiation-induced HDAC activity, resulting in sustained DNA damage, prolonged G2/M arrest, and enhanced apoptosis. Mechanistic analyses revealed that radiosensitisation was associated with RAD51 downregulation, inhibition of DNA-PKcs activity, modulation of p53 phosphorylation, and attenuation of SUMOylation-dependent DNA repair signalling. Transcriptomic profiling underscored cell-cycle regulation and DNA-repair pathways in response to combination treatment. Meanwhile, proteomic data implicated the modulation of SUMOylation as a central mediator of the observed radiosensitisation. Importantly, in physiologically relevant 3D spheroid models derived from patient meningioma specimens, the combined treatment synergistically augmented cell death, supporting the translational potential of this therapeutic strategy. Overall, this work establishes dacinostat as a promising radiosensitiser that compromises DNA repair fidelity and moduflates SUMOylation-associated pathways, thereby enhancing efficacy in treatment-resistant meningiomas. These findings provide a compelling preclinical basis to advance dacinostat into clinical assessment for the management of aggressive meningioma.

## Introduction

Meningiomas are the most prevalent intracranial tumours, constituting approximately 37.6% of primary brain neoplasms [1]. These tumours arise from the arachnoid cap cells and dural border cells found within the meninges [1]. The 2021 World Health Organisation (WHO) classification of CNS tumours advances meningioma characterisation by integrating molecular features with traditional grading, improving risk stratification. Meningiomas are classified into three grades: grade 1 (benign, 85–95%), with a low risk of recurrence; grade 2 (atypical, 5–10%); and grade 3 (anaplastic, 1–5%), which exhibit higher mitotic activity and more aggressive behaviour [2]. The classification incorporates molecular alterations, such as pTERT promoter mutations and CDKN2A/B homozygous deletions, linked to grade 3 meningiomas and a higher risk of recurrence [2].

Initial management of asymptomatic meningiomas typically involves observation, whereas symptomatic cases are predominantly treated through surgical resection aimed at maximal removal, especially for grade 1 meningiomas [3]. The extent of resection impacts prognosis, with gross total resection correlating with improved outcomes. However, a gross total resection is not always applicable, making adjuvant radiotherapy important, particularly for higher-grade or recurrent tumours [3]. Unfortunately, radiotherapy has limitations. Zollner et al. examined recurrence patterns following radiotherapy in 20 patients with high-grade meningiomas, reporting that 3 patients experienced local recurrence within the confines of the previous radiotherapy, highlighting in-field recurrence risk [4]. Moreover, radiotherapy can cause long-term health-related quality-of-life issues, necessitating careful consideration of its benefits versus harms [5]. Currently, chemotherapy remains ineffective for meningioma, as clinical trials have not shown significant improvements in patient outcomes [6].

HDACs are often overexpressed in various cancers, including meningiomas, where they restrain tumour suppressor genes and promote oncogenic pathways [7, 8]. Inhibiting HDACs shows promise in cancer therapy by modulating gene expression and inducing cell cycle arrest, differentiation, and apoptosis [9, 10]. Despite their therapeutic potential, HDACis exhibit limited cytotoxicity as monotherapies, especially in solid tumours, limiting their efficacy. Consequently, rational combination strategies with other targeted treatments or modalities are being actively explored to enhance therapeutic outcomes while minimising toxicity [11]. Our previous research indicates HDAC6 plays a role in meningioma growth and resistance, and its inhibition enhances radiotherapy by increasing DNA damage and reducing cell survival [8]. Currently, three FDA-approved HDACis, vorinostat, romidepsin, and belinostat, are used for haematological malignancies [12], but their effectiveness against meningioma has yet to be established. Panobinostat, initially approved by the FDA in 2015 for multiple myeloma, was withdrawn in 2021.

An integrative molecular grouping study on meningioma identified vorinostat as a potential therapeutic target for pathways upregulated in proliferative (MG4) meningiomas [13]. It selectively reduced the viability of MG4 patient-derived cell lines compared to those from other molecular groups [13], highlighting HDACis’s potential for treating aggressive meningiomas. HDACis, like panobinostat, dacinostat, and HC toxin, also decreased viability across various meningioma cultures, regardless of grade, recurrence, prior radiation therapy, or gender [7]. Currently, REC-2882 is undergoing Phase 2/3 clinical trials (NCT05130866), has shown favourable tolerability in patients with NF-2 mutated meningiomas and promising effects on slowing tumour progression in early-phase clinical trials [14, 15].

Dacinostat, also called NVP-LAQ824, inhibits DNA synthesis, triggers cell cycle arrest, and induces apoptosis in cancer cells [16]. We demonstrate that, among other FDA-approved HDACis, dacinostat most effectively reduces meningioma cell viability with radiation. Building on this finding, our investigation focused on two key objectives: first, whether a low nanomolar concentration might provide a more favourable therapeutic index, balancing efficacy with tolerability, given that dacinostat’s clinical use is limited by dose-dependent toxicity and potential resistance [17–19]. Secondly, we aimed to understand HDAC-mediated radioresistance, including DNA damage repair, cell cycle regulation, and cell death; comprehending these processes is crucial for developing strategies to overcome radioresistance.

In our previous study, we presented that radiation induces a specific class of HDAC expression that contributes to treatment resistance in meningioma [8]. In this study, we demonstrated that targeting this process with dacinostat presents a promising strategy to enhance therapeutic efficacy by increasing DNA damage, sustaining cell cycle arrest, and promoting apoptosis. Furthermore, we show that these effects are mechanistically mediated through the coordinated regulation of proteins involved in DNA damage response and cell cycle control. Moreover, global proteomic analysis further suggests that the radiosensitisation effect of dacinostat is likely driven by modulation of SUMOylation-related pathways. The enhanced efficacy of the combination treatment was consistently observed in both conventional 2D cultures and physiologically relevant 3D spheroids of meningioma, underscoring its translational potential.

## Results

### Dacinostat reversed radiation-induced HDAC activity and synergistically reduced cell survival and DNA replication

**Fig. 1:**
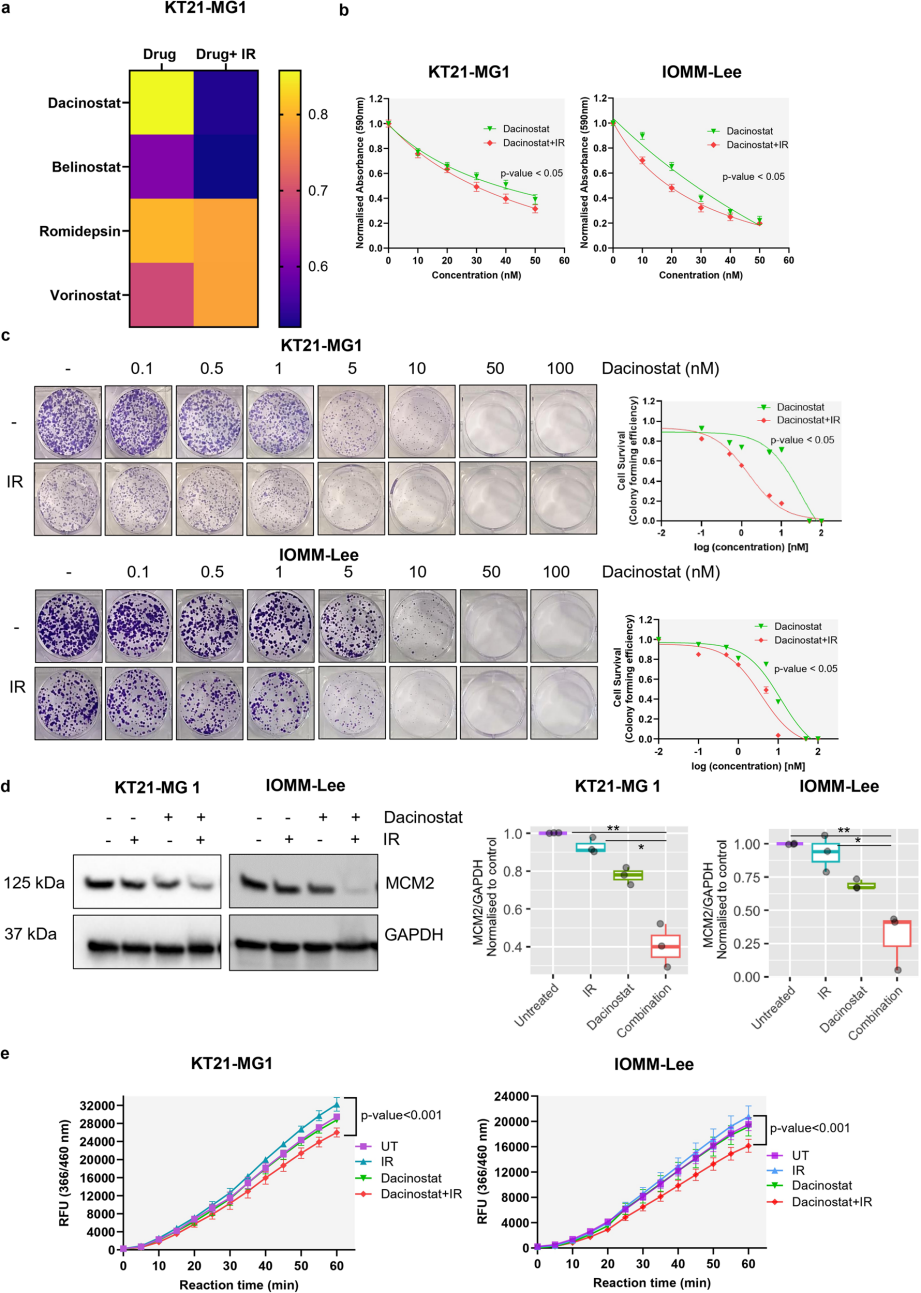
The pairing of dacinostat with IR reduces proliferation and counteracts IR-triggered HDAC activity. **a** The MTT assay showed that combining dacinostat with 4 Gy significantly reduced viability (*p* < 0.01) compared to other FDA-approved HDACis. **b** Cell viability decreased with dacinostat (10–50 nM) and IR (4 Gy for KT21-MG1 and 2 Gy for IOMM-Lee) for 96 h versus IR alone (*n* = 3, mean with SEM, paired t-test). **c** A representative image of the colony formation assay alongside the survival graph showed decreased cell survivability following the combination treatment (*n* = 3, mean with SEM, paired t-test). **d** Western blot analysis showed that MCM2 protein expression decreased with the combination treatment compared to untreated and single treatments (*n* = 3, mean ± SD, one-way ANOVA, Kruskal-Wallis). **e** HDAC activity assay revealed that IR-enhanced HDAC activity is reversed when combined with dacinostat (*n* = 3, mean with SEM, paired t-test).

The efficacy of dacinostat in combination with ionising radiation (IR) was assessed and directly compared to that of FDA-approved HDACis. In the KT21-MG1 cell line, the dacinostat-IR combination significantly reduced cell viability (*p* < 0.0001), outperforming inhibitory effects compared to other FDA-approved HDACis (Fig. 1a, Supplementary Fig. S1a, b). Dacinostat, a pan-HDACi, decreased the expression of HDACs from multiple classes while simultaneously increasing the histone H3 acetylation (Supplementary Fig. S1c), consistent with reflecting the inhibition of histone deacetylase activity and the resulting global histone hyperacetylation [20]. Furthermore, dacinostat increased cleaved PARP levels, a hallmark of apoptosis, compared with other HDACis, including REC-2882 (Supplementary Fig. S1c). Thus, we chose dacinostat for further analysis. We assessed the viability of immortalised cell lines at various concentrations of dacinostat in combination with irradiation, observing a notable decrease in cell viability in KT21-MG1 and IOMM-Lee (Fig. 1b); however, no change was observed in NCH93 (Supplementary Fig. S1d). The colony formation assay showed a significant reduction in survival in both KT21-MG1 and IOMM-Lee cells after combined treatment with dacinostat and radiation, compared with radiation alone (Fig. 1c). Radiation potentiated dacinostat-induced cytotoxicity in IOMM-Lee cells (2 Gy), with an enhancement ratio of 1.53 at 5 nM. In KT21-MG1 cells (4 Gy), the combination produced a stronger radiosensitising effect, with an enhancement ratio of 2.43 at 5 nM. The survival curves indicated IC_50_ values of 11.49 nM and 36.96 nM for IOMM-Lee and KT21-MG1, respectively, indicating that IOMM-Lee is more sensitive to dacinostat than KT21-MG1. MCM2 is a crucial marker for DNA replication and cell proliferation. MCM2 expression was significantly decreased after combination treatment in all three cell lines, KT21-MG1, IOMM-Lee, and NCH93 (Fig. 1d and Supplementary Fig. S1e), correlating with reduced colony formation. These findings suggest impaired proliferative capacity due to enhanced therapeutic response.

In our previous report, we demonstrated that radiation augments HDAC6 expression; however, co-treatment with radiation and the HDAC6 inhibitor, Cay1063, reduced this radiation-induced upregulation [8]. Given that elevated HDAC activity and decreased histone acetylation are critical determinants of chromatin condensation and the radioresistance phenotype [8, 21], we next investigated global HDAC activity. Notably, we observed that radiation-induced HDAC activity was significantly attenuated by dacinostat treatment (Fig. 1e). Furthermore, the histone H3 acetylation level was increased in a dose-dependent manner (Supplementary Fig. S1f). Overall, this suggests that the combination of dacinostat with radiation diminishes the proliferative capacity of meningioma cells and decreases the radiation-induced HDAC activity.

### Pre-treatment with dacinostat enhanced IR-induced DNA damage by inhibiting the DNA repair kinases

**Fig. 2:**
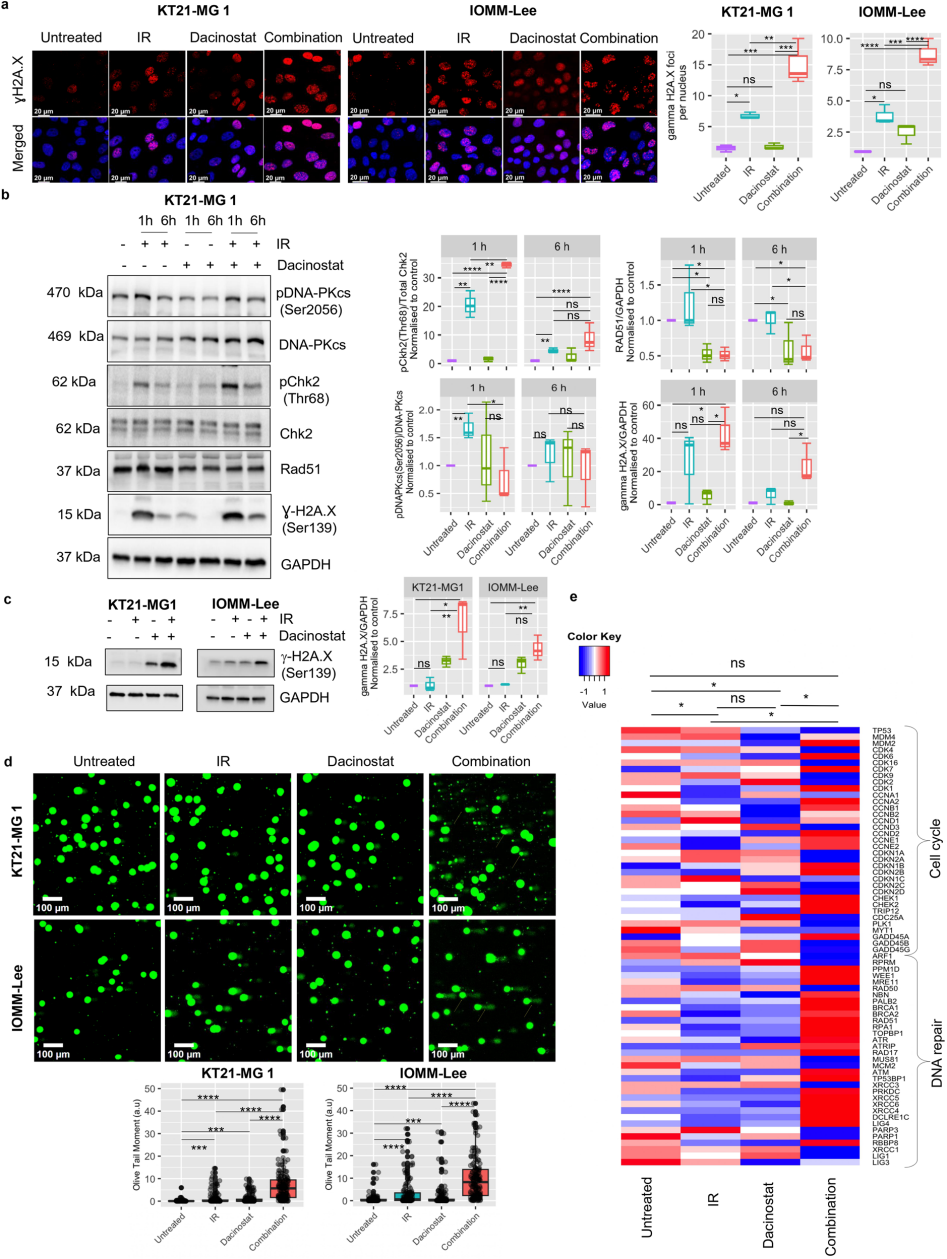
Dacinostat with IR alters DNA repair protein expression and boosts IR-induced DNA damage. **a** γH2A.X foci expression was observed 4 h after IR (4 Gy for KT21-MG1 and 2 Gy for IOMM-Lee), showing greater enhancement when IR was combined with dacinostat treatment (*n* = 3, mean with SD, one-way ANOVA, Tukey). **b** A western blot image shows protein regulation related to the cell cycle and DNA repair at 1 and 6 h post-IR. Protein analysis revealed that Chk2 activation significantly rose after combination treatment at 1 h but decreased more slowly than with IR alone. Prolonged γH2A.X expression occurred, while DNA-Pkcs activation and RAD51 expression lessened with the combination treatment. (*n* = 3, mean with SD, one-way ANOVA, Kruskal-Wallis). **c** The expression of γH2A.X remained significant even 24 h post-IR following combination treatment compared to controls (*n* = 3, mean with SD, one-way ANOVA, Kruskal-Wallis). **d** The alkaline comet assay conducted 6 h after IR showed increased OTM, indicating damaged DNA, following the combination treatment compared to the controls (*n* = 3, mean with SD, one-way ANOVA, Kruskal-Wallis). **e** The transcriptomic analysis heatmap revealed significant regulation of cell cycle and DNA repair genes following combination treatment, as compared to single treatments (one-way ANOVA, Kruskal-Wallis).

To evaluate how dacinostat affects IR-induced DNA damage, we analysed γ-H2AX foci, a marker of double-strand breaks (DSBs), 4 h after irradiation with 4 Gy (KT21-MG1) or 2 Gy (IOMM-Lee) (Fig. 2a). Cells were pre-treated with dacinostat (36 nM for KT21-MG1, 11 nM for IOMM-Lee) for 24 h prior to irradiation and maintained in the presence of the drug during the subsequent 4-hour post-irradiation period. The analysis showed that dacinostat alone did not significantly increase γ-H2AX foci compared to untreated controls. In contrast, radiation alone caused averages of 6.74 ± 0.30 (KT21-MG1) and 3.80 ± 0.44 (IOMM-Lee) foci/nucleus. Notably, the combination treatment significantly increased γ-H2AX foci to 15.05 ± 2.13 (KT21-MG1) and 8.73 ± 0.64 (IOMM-Lee) foci/nucleus, indicating synergistic DNA damage. To further investigate the kinetics of DNA damage, we assessed γ-H2AX expression at 1 and 6 h (Fig. 2b and Supplementary Fig. S2a) and 24 h (Fig. 2c). While radiation alone induces γ-H2AX expression, it appears to be repaired within 24 h (Fig. 2c), suggesting efficient DNA repair over time. Nonetheless, the combination treatment keeps γ-H2AX significantly elevated, suggesting dacinostat prolongs IR-induced γ-H2AX expression. Although in IOMM-Lee, the γ-H2AX levels after combination treatment are similar to radiation alone at 1 and 6 h (Supplementary Fig. S2a), sustained γ-H2AX expression was observed at 24 hours (Fig. 2c). Because γ-H2AX expression specifically identifies DSBs, we applied the alkaline comet assay to include single-strand DNA breaks (Fig. 2d). The OTM significantly increased with combination treatment compared to IR alone, with a 6.7-fold increase observed in KT21-MG1 cells and a 2.8-fold increase in IOMM-Lee. Transcriptomic analysis reveals significant regulation of genes involved in the cell cycle and DNA repair following combination treatment in KT21-MG1 (Fig. 2e), but not in IOMM-Lee (Supplementary Fig. S2b). Given increased DNA damage, we next investigated DNA repair proteins. Protein data from Fig. 2b and Supplementary Fig. S2a show that Ckh2 activation (pCkh2 Thr68), vital in DNA damage response, increased following combination treatment in all three cell lines, with delayed reversal compared to radiation alone. Meanwhile, activation of DNA-Pkcs (phosphorylation at Ser 2046), crucial in non-homologous end joining (NHEJ), increased after radiation, but was negated when combined with dacinostat in KT21-MG1 and NCH93; IOMM-Lee showed no significant change (Supplementary Fig. S2a). Additionally, the expression of RAD51, a key protein in homologous recombination (HR), decreased with dacinostat alone in KT21-MG1 and IOMM-Lee, but a synergistic reduction was observed in NCH93. Overall, pre-treatment with dacinostat before radiation enhances DNA damage by downregulating repair proteins, affecting HR and NHEJ, with varying effects by cell line and radiation dose.

### Pre-treatment with dacinostat prior to radiotherapy altered cell cycle distribution and induced p53-associated apoptosis

**Fig. 3:**
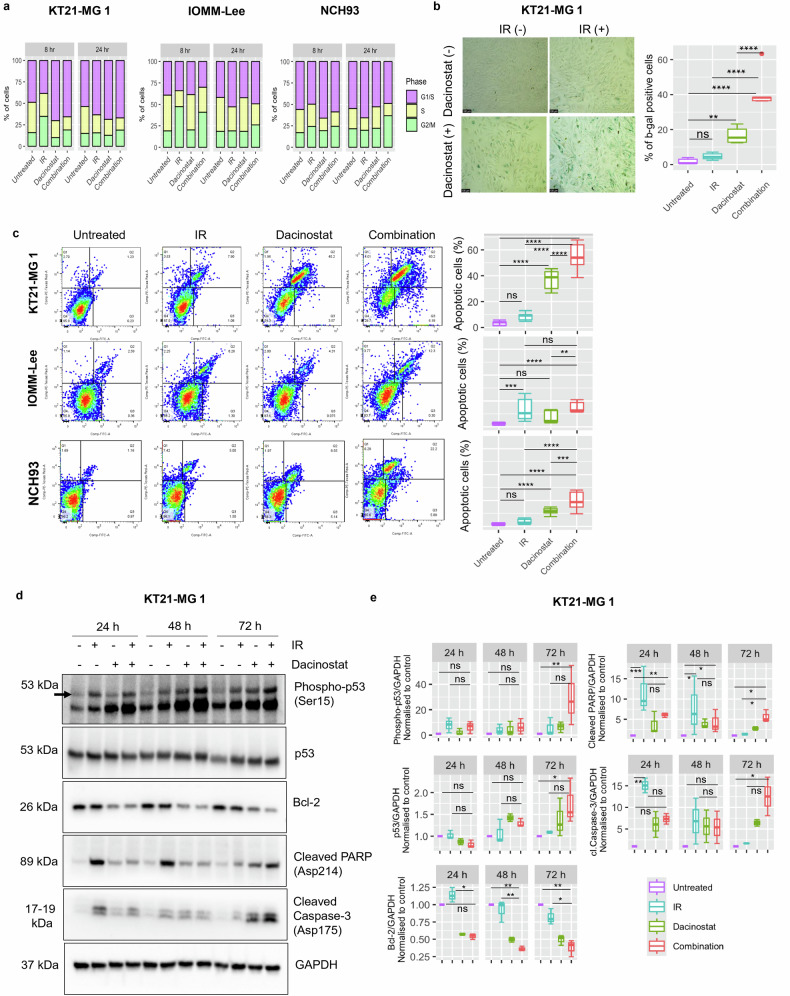
The combination of dacinostat and IR extends the cell cycle arrest, ultimately resulting in cell death. **a** The cell cycle analysis at 8 and 24 h post-IR (4 Gy for KT21-MG1 and NCH93, and 2 Gy for IOMM-Lee) showed that combination treatment maintained G1/S arrest in KT21-MG1, while extended G2/M arrest was observed in IOMM-Lee and KT21-MG1 at 24 h post-IR (Detailed analysis in Supplementary Fig. S3a, *n* = 3, mean with SD, one-way ANOVA, Tukey). **b** β-galactosidase staining 4 days after IR indicated an increase in senescence in cells following combination treatment (*n* = 3, mean with SD, one-way ANOVA, Tukey). **c** Annexin-V/PI flow cytometry showed that cells pretreated with dacinostat before IR undergo maximum apoptosis at 72 h post-IR (4 Gy for KT21-MG1 and NCH93, and 2 Gy for IOMM-Lee) (*n* = 3, mean with SD, one-way ANOVA, Tukey). **d** An representative image of the western blot displays the detection of Phospho-p53 (Ser15), total p53, and apoptosis indicators at various time points post-IR. **e** Protein quantification reveals that cells treated with the combination sustained high phospho-p53 (Ser15) and p53 levels, peaking at 72 h post-IR, which aligns with the maximum apoptosis. Cleaved PARP and cleaved caspase 3 did not match the apoptosis assay at 24 and 48 h, but their expression was higher than controls at 72 h post-IR, while Bcl-2 was downregulated after combination treatment (*n* = 3, mean with SD, one-way ANOVA, Kruskal-Wallis).

Given that transcriptomic analysis indicated that genes associated with the cell cycle are regulated after combination treatment (Fig. 2e), we analysed the cell cycle distribution at 8- and 24-hours post-IR to investigate cell cycle progression (Fig. 3a and Supplementary Fig. S3a). Radiation triggers a reversible G2/M cell cycle arrest, and our previous study demonstrated that inhibiting HADC6 leads to a non-reversible G2/M arrest [8, 22]. Cells treated with IR displayed increased G2/M arrest at 8 h. Unexpectedly, dacinostat alone induced G1/S arrests in KT21-MG1 and NCH93, but not in IOMM-Lee. Following a 24-h combination treatment, the KT21-MG1 showed significant G2/M arrest alongside G1/S arrest. NCH93 predominantly showed a G2/M arrest post-radiation, indicating a transition from G1/S to G2/M arrest at 24 h. Additionally, IOMM-Lee also demonstrated a prolonged G2/M arrest after combination treatment (Fig. 3a and Supplementary Fig. S3a). As prolonged cell-cycle arrest following radiation can indicate senescence [23], we performed β-galactosidase staining to identify senescent cells. In KT21-MG1, radiation alone did not significantly affect senescence; however, pretreatment with dacinostat synergistically increased senescence (23.4-fold, 9.1-fold, and 2.5-fold increase compared to UT, IR alone, dacinostat alone, respectively; Fig. 3b), consistent with dacinostat prolonging the G1/S phase (Fig. 3a). In contrast, in the IOMM-Lee, dacinostat neither induced senescence on its own nor enhanced radiation-induced senescence (Supplementary Fig. S3b). Since persistent senescence can lead to cell death, we conducted Annexin/PI staining, which showed a significant increase in apoptosis in all three cell lines with the combination treatment (Fig. 3c and Supplementary Fig. S3c). A time-dependent increase was observed in KT21-MG1 and IOMM-Lee, with peak apoptosis at 72 h. NCH93, assessed only at the 72-h time point, also showed a robust apoptotic response to the combination treatment. In human glioma cells, the phosphorylation of p53 at Ser15 and Ser20 triggers apoptosis by increasing pro-apoptotic genes [24]. Similarly, phospho-p53 (Ser 15) and total p53 peaked at 72 h after combination treatment (Fig. 3d, e and Supplementary Fig. S4a, b), coinciding with maximum apoptosis (Fig. 3c). Additionally, cleaved PARP also increased synergistically at 72 h, matching Annexin V/PI results. However, no significant elevation occurred at 24 or 48 h after IR and dacinostat treatment, indicating a delayed apoptotic response (Fig. 3d, e, Supplementary Figs. S3c, S4a–c). Concurrently, Bcl-2 and survivin, anti-apoptotic proteins, were also decreased. Altogether, the combination treatment extended cell-cycle arrest, with phosphorylated p53 inducing more apoptosis than radiation or drug treatment alone.

### Combination therapy shows reduced sensitivity in human meningeal cells (HMC) while remaining effective in radioresistant meningioma models

To assess the selectivity of the combination treatment, experiments were performed in human meningeal cells (HMC). CellTiter-Glo analysis showed that the combination of dacinostat and radiotherapy did not significantly reduce the viability of HMCs, despite a significant reduction in viability observed in IOMM-Lee cells (Supplementary Fig. S5a). Consistently, Western blotting revealed no basal expressions of BCL2, RAD51, phosphorylated p53 in HMC compared with IOMM-Lee. Low levels of SUMO1 and survivin were detectable in HMC and showed a decreasing trend following treatment (Supplementary Fig. S5b).

To examine effects in radioresistance, radioresistant (RR) KT21-MG1, and IOMM-Lee models were generated and confirmed by cell survival assays (Supplementary Fig. S5c). RR cells exhibited reduced sensitivity to the combination, though relative inhibitory effects persisted. Western blot analysis shows RAD51 was significantly downregulated following radiotherapy alone and in combination compared to untreated RR, indicating DNA repair pathways remain responsive. Phosphorylated p53 increased in RR cells after radiotherapy alone or combination treatment compared to untreated RR, showing active DNA damage signalling. Furthermore, the expression of anti-apoptotic proteins, including survivin and BCL2, showed reduced trends following combined dacinostat and radiotherapy treatment in RR cells compared with untreated RR controls (Supplementary Fig. S5d), suggesting that this combination strategy also effective in radioresistant models.

### Reduced SUMOylation enhances radiosensitisation following the combination of dacinostat and radiation

**Fig. 4:**
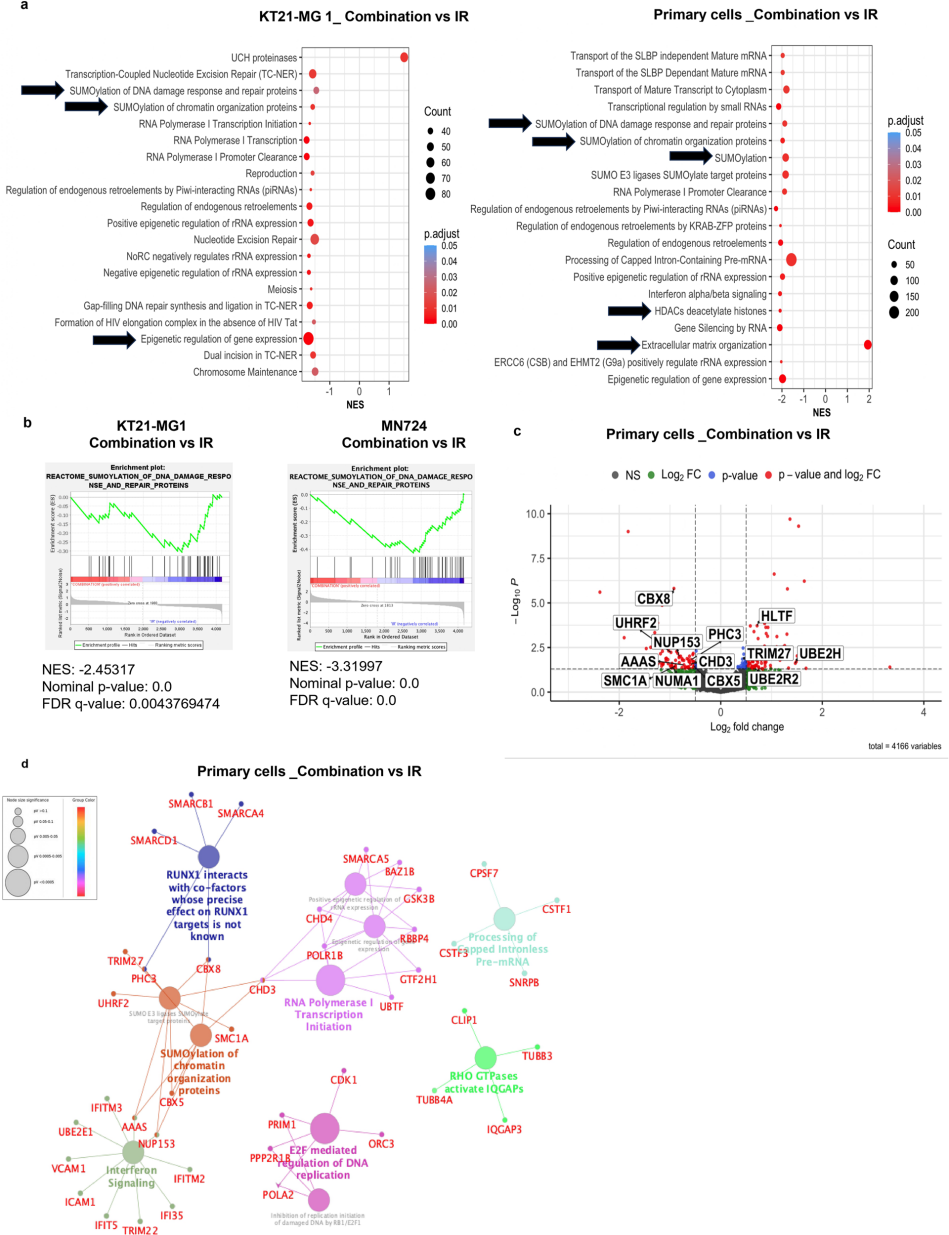
SUMOylation and its related pathways are downregulated following the combination treatment. **a** The dot plot from the ReactomePA comparing combination treatment and IR (4 Gy for KT21-MG1 and 12 Gy for primary cells) shows the top 20 enriched pathways. Dot colour indicates expression change significance (p adjusted), and size represents the number of enriched proteins. **b** Enrichment plots from gene set enrichment analysis (GSEA) demonstrate the decreased SUMOylation of DNA damage response and repair proteins following combination treatment in KT1-MG1 and MN724. NES, Normalised enrichment score. **c** The volcano plot of DEGs from the DEseq2 analysis of primary cells showed significant downregulation of SUMOylation-related proteins, while a few ubiquitination-related proteins were upregulated. **d** The outcomes from the DEseq2 analysis of primary cells and the ReactomePA results obtained through the ClueGO/Cluepedia plug-in were visualised in Cytoscape, using a cutoff value of *p* < 0.05. Terms grouped with a kappa score of≥ 0.4 are depicted as nodes in various colours.

We conducted a global proteomics analysis to gain a more in-depth understanding of protein-level changes following combination treatment. We compared the combination treatment against the IR condition in both immortalised meningioma cell lines and primary patient-derived cells. The top 20 significantly regulated pathways, as identified through proteomic analysis, exhibited a reduction in those associated with SUMOylation, along with decreases in HDACs and epigenetic regulation of gene expression following combination treatment in KT21-MG1 and primary cells (Fig. 4a); this effect was not observed in IOMM-Lee (Supplementary Fig. S6a). Gene set enrichment analysis further supported this finding, indicating decreased SUMOylation of DNA damage response and repair proteins under combination treatment (Fig. 4b). A volcano plot comparing treatment conditions (Combination vs IR) across 3 primary cell types identified 756 proteins as significantly regulated in response to the combination treatment (Fig. 4c). Notably, several ubiquitination-related proteins, including UBE2H, UBE2R2, HLTF, and TRIM27, were significantly upregulated. Conversely, proteins associated with SUMOylation, including CBX8, CBX5, NUP153, UHRF2, PHC3, AAAS, CHD3, SMC1A, and NUMA1, showed a significant decrease. This similar pattern was observed in KT21-MG1, wherein proteins associated with ubiquitination were markedly upregulated, and the SUMOylation-related protein, SUMO1, was significantly downregulated (Supplementary Fig. S6b). A pathway annotation network constructed using ClueGo (*p* < 0.05) from significantly regulated proteins revealed functional clusters related to SUMOylation, particularly in proteins involved in chromatin organisation and E2F-mediated regulation of DNA replication (Fig. 4d). Additional ClueGo analysis was performed without applying the *p* < 0.05 threshold, as the input consisted exclusively of proteins that were already identified as significantly differentially regulated in the proteomic analysis. Proteins associated with SUMOylation, RNA polymerase I transcription inhibition, mitotic G1 phase and G1/S transition, and RHO GTPase signalling formed distinct functional clusters (Supplementary Fig. S6c). This approach enabled the detection of coordinated pathway-level trends that may not reach individual statistical significance after multiple-testing corrections. Additionally, the expression of the SUMO1 protein in KT21-MG1 exhibited a synergistic decrease following the combination treatment (Supplementary Fig. S6d). In summary, the results indicate that the combination of dacinostat and radiation has a significant effect on SUMOylation and its associated pathways, compared to IR alone.

### Dacinostat pre-treatment synergistically radiosensitises 3D spheroids derived from cell lines and patients’ meningioma cells

**Fig. 5:**
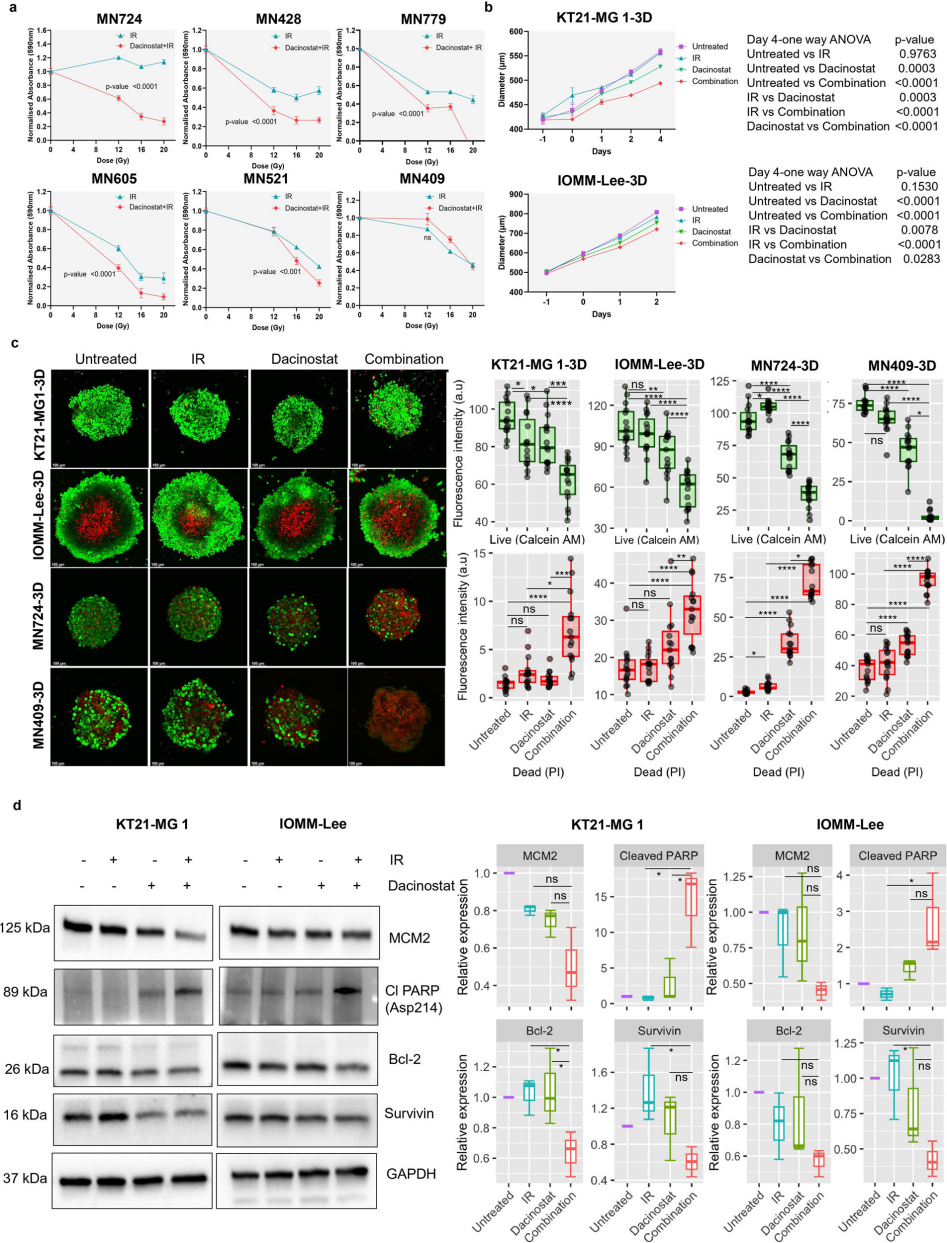
The combination treatment reduced growth and increased cell death of 3D spheroids from primary and immortalised cell lines. **a** Except for MN409, the viability of primary cells pre-treated with dacinostat before IR (12 Gy) significantly decreased compared to the radiation control (*n* = 3, mean with SEM, Mann-Whitney test). **b** The growth kinetics revealed that spheroids from immortalised cells decreased after combination treatment compared to controls (*n* = 3, mean with SEM, one-way ANOVA, Tukey). **c** Live and dead assays on spheroids demonstrated that pre-treating with dacinostat before IR (4 Gy for KT21-MG1, 2 Gy for IOMM-Lee and 12 Gy for primary cells) synergistically enhances cell death in spheroids (*n* = 3, mean with SD, one-way ANOVA, Tukey). **d** Western blot analysis of spheroid lysates indicates a synergistic increase in cleaved PARP following combination treatment, whereas MCM2 and the anti-apoptotic proteins Bcl-2 and survivin were reduced (*n* = 3, mean with SD, one-way ANOVA, Kruskal-Wallis).

Given that patient-derived cells better preserve intra-tumoural heterogeneity and mimic the physiological characteristics of the tumour than conventional cell lines, we assessed the efficacy of dacinostat and radiation in patient-derived meningioma samples. We tested dacinostat on primary cells, determining IC_50_ for 6 different primary cells to guide dose selection (Supplementary Fig. S7a). Patient-derived meningioma cells showed increased radiation resistance, requiring higher doses than those used for immortalised cell lines. Five of six primary cell models exhibited a synergistic reduction in viability following combined treatment, whereas one (MN409) showed only an additive effect (Fig. 5a). Spheroids replicate key features of in vivo tumours, including intercellular contact, nutrient gradients, and hypoxia, contributing to treatment resistance. Therefore, we employed a 3D spheroid model of meningioma, established in our previous study [25], derived from cell lines KT21-MG1 and IOMM-Lee, as well as early passages of primary cells (P3 for MN724 and P4 for MN409). To understand the impact of combination treatment on spheroid proliferation, we conducted a growth kinetic analysis. Spheroids derived from immortalised cell lines exhibited markedly reduced growth after combination treatment (Fig. 5b and Supplementary Fig. S8a), while spheroids derived from patients did not show growth differences (Supplementary Fig. S8b), confirming our previous findings [25]. Viability assays with calcein AM and PI revealed that the combination treatment synergistically decreased live and increased dead cells compared to controls and IR alone (Fig. 5c). Interestingly, IR alone significantly increased calcein AM intensity in MN724 compared to untreated cells, aligning with 2D viability results (Fig. 5a). Although MN409 showed only additive effects in monolayer (Fig. 5a), it exhibited a synergistic reduction in spheroids, highlighting that the combinational impact becomes more pronounced in an advanced 3D culture system. Finally, western blot analysis using protein lysates from spheroids indicated that the combination treatment lowered MCM2, Bcl-2, and Survivin, and increased cleaved PARP (Fig. 5d), consistent with the 2D data (Fig. 1d, 3d, Supplementary Figs. S1c, S4a). These molecular changes collectively support that the combination treatment is more effective than radiation alone in suppressing tumour growth by both inhibiting proliferation and promoting apoptosis. Overall, the combination of dacinostat and radiation enhanced therapeutic efficacy in 3D spheroid models, mirroring the synergistic effects observed in monolayer cultures.

## Discussion

In this study, we investigated the potential of the pan-HDACi dacinostat to enhance the efficacy of radiotherapy in meningioma models. We found that dacinostat combined with radiation synergistically reduced cell viability to a greater extent than combinations involving FDA-approved HDACis (Fig. 1a). While REC-2282 represents a major clinical milestone as the first HDAC inhibitor to enter trials for meningioma (NCT05130866), our comparative data (Supplementary Fig. S1a, b) suggest that its radiosensitising potential may be more modest than that observed with dacinostat. This discrepancy highlights a potential translational gap between current clinical candidates and the high-potency epigenetic modulation required for robust radiosensitisation. Despite the discontinuation of dacinostat after Phase II trials due to cardiotoxicity risks, including QT prolongation [19, 26], its broad-spectrum antitumour activity warrants further investigation, particularly at lower doses or in combination with other treatments [17]. The next steps in the translational pathway should focus on identifying the specific molecular signatures, such as isoform-specific inhibition or chromatin remodelling patterns, that allow dacinostat to outperform its clinical counterparts. Future investigation is required to determine whether next-generation epigenetic modifiers, or PROTAC-based degraders, can achieve dacinostat-like synergy while maintaining an acceptable safety profile for intracranial application. Furthermore, given dacinostat’s documented synergy in other malignancies like non-small cell lung cancer via impaired DNA repair [27], our findings in both 2D monolayer and 3D spheroid cultures (Figs. 1, 3, 5) provide a strong rationale for prioritising more potent HDAC inhibition in future meningioma combination strategies.

Notably, this radiosensitising effect stems from reduced DNA repair, partly through modulation of the SUMOylation pathway, and offers novel mechanistic insights and therapeutic avenues to overcome resistance in meningioma (Figs. 2, 4). Radioresistance remains a major obstacle in the treatment of high-grade meningioma, which is characterised by frequent recurrence and limited responsiveness to conventional radiotherapy. Several contributing factors have been proposed, including NF2 loss-of-function mutations under hypoxic conditions, differential microRNA expression, and HDAC6 overexpression [8, 28, 29]. A recent integrative molecular classification of meningiomas identified MCM2 enrichment in MG4 meningiomas, indicating an aggressive phenotype [13]. Given our study’s focus on high-grade meningiomas, it is crucial to investigate MCM2 regulation following combination treatment. As expected, combination treatment decreased MCM2 expression (Fig. 1d and Supplementary Fig. S1c) and, simultaneously, reduced cell survival (Fig. 1c). Furthermore, we found that radiation alone increases HDAC activity, whereas pre-treatment with dacinostat significantly reduces the IR-induced HDAC activity (Fig. 1e), thereby maintaining DNA DSBs, as indicated by persistent γ-H2AX expression (Fig. 2c).

The activation of Chk2 by HDACi has been reported to contribute to radiosensitisation by inducing a DNA damage response [30]. Consistent with this, we observed increased Chk2 activation following combination treatment with dacinostat and radiation (Fig. 2b and Supplementary Fig. S2a). Cell line-specific differences were also evident. Dacinostat alone induced G1/S arrest in KT21-MG1 and NCH93, indicating a clinically meaningful cell-cycle perturbation, given the therapeutic targeting of the CDK4/6-mediated G1/S transition in meningioma, with agents such as abemaciclib (NCT05940493, NCT02523014) and ribociclib (NCT02933736) currently under clinical evaluation [31]. In contrast, IOMM-Lee cells exhibited a prolonged radiation-induced G2/M arrest following dacinostat treatment (Fig. 3a and Supplementary Fig. S3a), indicating cell-line-specific cell-cycle responses. This discrepancy is likely due to the homozygous deletion of CDKN2A/B in IOMM-Lee, which encodes p16(INK4A) and p14(ARF), suggesting this mutation impedes G1/S arrest [32, 33]. Supporting this, studies in lung cancer models have shown that tumours with deleted CDKN2A fail to respond to HDACis, unlike those with epigenetically silenced CDKN2A [34]. Since p16 (INK4A) plays a crucial role in inducing senescence [35], its absence may explain the lack of dacinostat-induced senescence in IOMM-Lee (Supplementary Fig. S3b).

Simultaneous inhibition of Rad51 and DNA-PKcs may enhance radiosensitisation by disrupting both DSB repair pathways: HR and NHEJ [36, 37]. Engagement of DNA repair pathways depends on both cell cycle phase and radiation dose. HR is preferentially active in G2 and is favoured at lower radiation doses [38, 39]. In our study, combination treatment induced a prolonged G2/M arrest, yet reduced RAD51 expression likely impaired HR despite the accumulation of cells in this repair-permissive phase. Specifically, KT21-MG1 and NCH93 cells received 4 Gy and showed decreased RAD51 expression and reduced DNA-PKcs activation following combination treatment (Fig. 2b and Supplementary Fig. S2a), suggesting disruption of both HR and NHEJ pathways. In contrast, IOMM-Lee, which received only 2 Gy, showed reduced RAD51 but unaltered DNA-PKcs activation, indicating preferential disruption of HR (Supplementary Fig. S2a). Together, these findings demonstrate that RAD51 downregulation and altered cell-cycle distribution act cooperatively, in a dose and genotype-dependent manner, to determine radiosensitisation.

The timing of p53 Ser15 phosphorylation after radiosensitisation influences cellular responses to DNA damage, varying across cell types and doses. Stewart-Ornstein et al. found that radiosensitive tissues exhibit prolonged p53 signalling post-IR, whereas resistant tissues show brief activation [40]. We observed similar p53 phosphorylation dynamics; prolonged after combination treatment, but only temporary with IR alone (Fig. 3d, e, Supplementary Figs. S4a, b, d, e). In IOMM-Lee, however, the dynamics were similar in both treatments (Supplementary Fig. S4e), aligning with no significant difference in cell death rates (Fig. 3c).

Proteomic analysis enabled the identification of post-translational modifications (PTMs) that are frequently overlooked at the transcriptomic level. Among these, SUMOylation is crucial in cancer therapy resistance by regulating transcription, DNA damage repair, and the cell cycle, making it a compelling target for improving radiotherapy efficacy [41]. Broad alterations in SUMO conjugation can occur during HDAC inhibition; however, these effects depend on both the specific SUMO paralog and the cellular context. For example, Class I HDACi increases SUMO1 conjugation of proteins in cardiomyocytes [42], while other HDACi treatment selectively inhibit Ubc9 acetylation, halting SUMOylation for certain targets [43]. To the best of our knowledge, no reports have yet indicated that inhibiting HDACs enhances radiosensitivity via downregulating SUMOylation-associated pathways. In KT21-MG1 and patient-derived meningioma cells, dacinostat and radiation together reduce SUMOylation-related pathways associated with DNA damage response and chromatin organisation (Fig. 4a, b). In contrast, IOMM-Lee shows no SUMOylation (Supplementary Fig. S6a), offering another explanation beyond the dynamics of p53 activation for the reduced effectiveness of this combination treatment in the 2D model, as indicated by cell death data (Fig. 3c and Supplementary Fig. 3b) compared to KT21-MG1 and NCH93.

Collectively, IOMM-Lee’s differential response likely results from CDKN2A/2B homozygous deletion, which is linked to poor prognosis and treatment resistance [44], as well as the lower radiation dose used. These findings highlight how tumour heterogeneity, including genetic and microenvironmental factors, affects treatment outcomes (Supplementary Table 7). Despite variable sensitivity across cell lines, our study expands understanding of HDACi-mediated radiosensitisation and reveals a novel role for SUMOylation in meningioma response to radiotherapy.

To enhance the translational relevance of the combination of dacinostat and radiation, we conducted experiments in both 2D monolayer and 3D spheroid models derived from primary meningioma cells. While patient-derived orthotopic xenografts (PDOX) are the most widely accepted in vivo model, establishing them in meningioma remains challenging. For instance, Zhang et al. reported the challenges in establishing PDOX models from meningioma samples, with only one out of nine engrafting successfully [45]. On the other hand, 3D meningioma spheroids retain key tumour characteristics, including morphological features, genomic alterations, and the tumour-associated immune microenvironment, such as macrophage populations, offering a more reproducible and accessible system [25]. Herein, we utilised early-passage patient-derived cells in 2D and 3D models to preserve in vivo properties. Research into the pharmacological profiling of 3D tumour models found that combinations such as Navitoclax and Carfilzomib showed greater synergy in 3D spheroid cultures than in 2D monolayers, likely due to the complex interactions and signalling pathways in 3D cultures [46]. Likewise, MN409 cells did not respond to the combination treatment in monolayer cultures but exhibited a synergistic reduction in viability in 3D spheroids (Fig. 5a, c). This reinforces the importance of 3D models, which better reflect complex cell interactions and more accurately capture treatment effects. This discrepancy may also be attributed to the treatment duration of seven days following the combination treatment in 3D spheroids, compared to four days in 2D cell cultures.

**Fig. 6:**
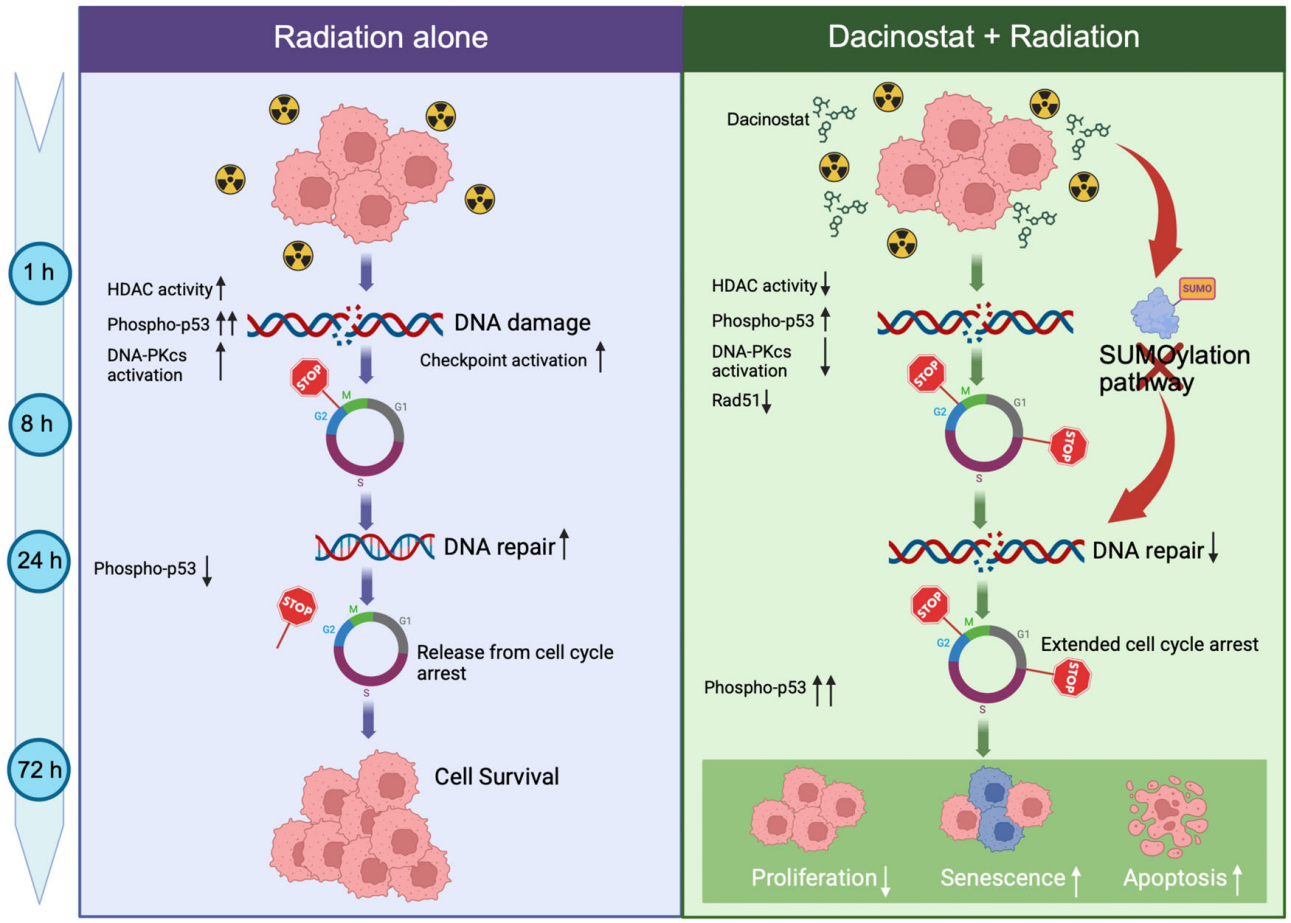
A schematic diagram depicting the mechanism of dacinostat-mediated radiosensitisation potentially involves temporal regulation of p53 phosphorylation and the SUMOylation pathway.

In summary, our study demonstrates that dacinostat is a promising candidate for use in conjunction with radiation therapy, without requiring high cytotoxic concentrations. Integrating dacinostat with IR resulted in reduced HDAC activity, enhanced DNA damage via modulation of SUMOylation-associated pathways, and decreased expression of DNA repair proteins. Furthermore, the combination therapy altered phospho-p53 dynamics, thereby facilitating cell death in both meningioma 2D settings and 3D spheroid models (Fig. 6), underscoring its potential to augment the efficacy of radiotherapeutic interventions.

## Materials and methods

All cell lines, reagents, instruments, and online tools utilised are documented in Supplementary Tables 1 and 2.

### Cell culture

The immortalised cell lines KT21-MG1, IOMM-Lee and NCH93 were cultured in high-glucose DMEM, supplemented with 10% FBS and without antibiotics. Grade 2 meningioma primary cells (MN724, MN428, MN779, MN505, MN521, and MN409) were maintained in a DMEM/F-12 nutrient mixture (1:1), supplemented with 20% FBS and 1% penicillin-streptomycin. The cells were incubated at 37 °C and 5% CO_2_ in a humidified incubator. The specifics of the immortalised cell lines used in this study are as previously described [8]. Cell line authentication was performed by short tandem repeat (STR) profiling (Eurofins Genomics). The IOMM-Lee cell line matched the published STR reference profile. For NCH93 and KT21-MG1, STR profiles were obtained; however, no reference profiles are currently available in public databases (e.g. Cellosaurus or DSMZ), precluding direct comparison. Nevertheless, cell line authentication analysis confirmed that each culture represented a single, unique cell line with no evidence of cross-contamination. Cells were tested for mycoplasma contamination and confirmed to be negative.

To model acquired radioresistance, IOMM-Lee and KT21-MG1 sublines (RR) were established using a chronic induction protocol. Parental cells were subjected to five cycles of radiation (specifically 2 Gy and 4 Gy per fraction, respectively) calibrated to account for their distinct baseline radiosensitivities. This approach resulted in a cumulative dose of 10 Gy for IOMM-Lee and 20 Gy for KT21-MG1, ensuring a robust selective pressure for the development of a radioresistant phenotype.

Primary meningiomas were obtained from patients at the University Hospital Plymouth NHS Trust and Southmead Hospital, Bristol. The University of Plymouth has a Brain Tumour Bank focused on low-grade or slowly growing brain tumour types. The National Research Ethics Committee granted ethical approval for the biobank, which is registered in the UK under reference numbers REC 19/SC 0267 and IRAS number 246 667. Informed consent was obtained in writing from all patients prior to the surgical procedures. The clinical, histopathological, and molecular characteristics of all patients included in this study are summarised in Supplementary Table 3.

### Ionising radiation

Immortalised cell lines and primary cells were subjected to X-ray exposure, administered at doses of 2 Gy for IOMM-Lee, 4 Gy for KT21-MG1 and NCH93 [8], and between 12 and 20 Gy for primary cells, using a linear accelerator (LINAC) (Varian Medical Systems, Palo Alto, CA, USA). The radiation administration was conducted by qualified radiologists, radiotherapy engineers, or clinical scientists within the radiotherapy department at Derriford Hospital, Plymouth, UK.

Radiation dosimetry was performed in collaboration with the clinical radiotherapy physics team using a routinely calibrated clinical LINAC. Prior to irradiation, dose calculations were carried out to confirm accurate dose delivery to in vitro samples, with experimental flasks and dishes provided in advance to enable dosimetric planning based on the exact sample geometry. Several irradiation configurations were evaluated, including anterior beam delivery with and without build-up material and posterior beam delivery. Dose homogeneity was assessed using dose-volume histograms (DVHs), and posterior beam irradiation provided the most homogeneous dose distribution across the cell culture volume (coefficient of variance ~2.6%) and was therefore selected for all experiments. Irradiation was performed using a 6 MV photon beam with the gantry at 180° and a 30 × 30 cm field size, with monitor units adjusted to deliver doses ranging from 2 to 20 Gy.

### Drug treatment

Multiple HDAC inhibitors, such as Dacinostat, Belinostat, Romidepsin, Panobinostat, Cay10603, SB939, TSA, REC-2282, and Vorambucil, were used to compare their efficacy in the meningioma cell line. The details of HDACis used are provided in Supplementary Table 1. Drug treatment began 24 h after cell plating and continued for 24 h before radiation exposure. Immortalised cells (11 nM for IOMM-Lee and 36 nM for KT21-MG1 and NCH-93) and primary cells were treated with the determined IC_50_ of dacinostat (Supplemental Fig. S5a).

### Cell viability analysis

MTT (3-(4,5-dimethylthiazol-2-yl)-2,5-diphenyltetrazolium bromide) assays were carried out as previously described [47]. Briefly, 3000 cells per well were seeded in 96-well plates and treated with the drug and radiation. MTT viability was assessed via MTT incubation, followed by DMSO addition, and absorbance was measured at 590 nm using a spectrophotometer (FLUORstar Omega, BMG Labtech, Ortenberg, Germany).

Cell viability was quantified using the CellTiter-Glo Luminescent Assay (Promega, Madison, WI) according to the manufacturer’s protocol and as previously described [25]. Briefly, 2000 cells per well were seeded in 96-well plates. To evaluate the efficacy of dacinostat in overcoming acquired resistance, the WT, and radioresistant (RR) lines were pre-treated with dacinostat (11 nM for IOMM-Lee; 36 nM for KT21-MG1) for 24 h prior to radiotherapy. Given the distinct proliferation kinetics of these models (specifically a doubling time of 20 h for IOMM-Lee compared to 28 h for KT21-MG1) as shown in our previous study [8], post-irradiation incubation periods were optimised to 2 and 4 days, respectively. This temporal adjustment ensured that the luminescent signal accurately reflected the treatment’s impact on each cell line’s specific growth trajectory.

### Colony formation assay

The colony formation assay was performed as previously described [48]. In summary, 1200 cells per well were seeded in a 6-well plate and incubated for 10 days (IOMM-Lee), or 14 days (KT21-MG1) following irradiation. Radiation doses were 2 Gy for IOMM-Lee and 4 Gy for KT21-MG1, as determined in our previous study [8]. After incubation, the media were discarded, and the wells were washed with distilled water prior to crystal violet staining to visualise colonies.

### HDACs enzyme activity assay

HDAC activity was assessed using an HDAC assay kit according to the manufacturer’s instructions. Nuclei from 1 × 10^7^ cells were isolated, extracted, sonicated for 30 s and centrifuged to yield a crude nuclear extract. Protein concentration was measured via the BCA assay kit. Reactions began by adding 10 µg of nuclear extract to wells containing HDAC assay buffer, substrate peptide, and developer. The plate was mixed at room temperature, and fluorescence intensity was recorded over 60 minutes, at 1-min intervals using an automated microplate reader (FLUOstar Omega), with excitation at 360 nm and emission at 460 nm.

### Western blotting

Cells and spheroids were lysed with RIPA buffer and Halt™ Protease and Phosphatase Inhibitor cocktail. Spheroids underwent three freeze-thaw cycles using liquid nitrogen and a 37 °C water bath, followed by sonication (XUBA1 Ultrasonic Bath, Grant Instruments, Cambridge, UK) for 2-min sessions twice, with a 1-min ice resting period in between. The protein lysate was collected after a 10-minute centrifugation at 13,000 rpm. Western blotting was performed as previously described [49]. Antibody details are listed in Supplementary Table 4.

### Immunofluorescence

Immunofluorescence was performed as previously described [48], with a minor modification mentioned in this section. Briefly, cells were fixed with 4% paraformaldehyde 4 h post-radiation (2 Gy and 4 Gy for IOMM-Lee and KT21-MG1). Antibody details are listed in Supplementary Table 4. Fluorescence images were captured with an SP8 confocal microscope (Leica Microsystems, Wetzlar, Germany).

### Comet assay

The comet assay followed the manufacturer’s instructions. In brief, 1500 cells were mixed with 60 µL of agarose and placed on a slide to solidify 24 h post-radiation. Cells were lysed and subjected to alkaline electrophoresis. The slides were rinsed, fixed with ethanol, air-dried, and stained with Vista Green DNA dye. Fluorescence images were captured at 20X magnification using an SP8 confocal microscope (Leica Microsystems). A minimum of 50 cells per condition were analysed using the CaspLab comet assay software to calculate the olive tail moment (OTM), which reflects the extent of DNA damage within each cell.

### Cell cycle analysis

Cells were collected and fixed in 70% cold ethanol overnight at −20 °C, then centrifuged at 400 × *g* for 10 min before being rinsed with cold PBS. Following that, the cells were treated with 200 μg/mL RNase for 2 h at 37 °C and stained with 5 µg/ml propidium iodide (PI) in PBS containing 0.1% Triton X-100 for 15 min in the dark prior to analysis. The cell cycle distribution was assessed using flow cytometry at 585 nm (BD Accuri™ C6, BD Biosciences, NJ, US).

### Annexin V assay

Apoptosis was measured using annexin V-FITC and PI staining. The detailed procedure has been described in our previous work [8, 48]. Cells were harvested, rinsed in PBS, and stained with 5 µg/ml propidium iodide in annexin V-FITC binding buffer [10 mM HEPES (pH 7.4), 140 mM NaCl, 2.5 mM CaCl_2_ for 15 min in the dark. Annexin V-FITC was added before flow cytometry (BD FACS Aria™ II, BD Biosciences).

### β-galactosidase (SA-gal) assay

Cellular senescence was measured using a β-galactosidase staining kit as per the manufacturer’s instructions (Cell Signalling Technology). Cells were fixed, rinsed, and stained and then incubated overnight at 37 °C without CO_2_. Images were captured at 20x objective using a DMi8 confocal system (Leica Microsystems).

### 3D spheroid growth response and viability assay

3D spheroids were cultured, and their growth response to treatment was measured as previously described [25, 50]. Spheroid viability was measured using a Live/Dead viability kit, following the manufacturer’s instructions with minor adjustments to detect membrane-compromised cells using PI.

### RNA-sequencing

Total RNA was extracted using the Direct-zol™ RNA MiniPrep kit following the manufacturer’s instructions. RNA purity and concentration were measured using the NanoPhotometer N60 (Implen, Munchen, Germany). RNA library preparation, transcriptome sequencing, and analysis were performed by Novogene Co., Ltd (Cambridge, UK). Genes with adjusted *p* < 0.5 and |log2(Fold Change) | > 1 were deemed differentially expressed. Lists of all significantly differentially expressed genes (DEGs) are provided in the Supplementary Table 5.

### Mass spectrometry analysis

To enrich the protein content, 50 µg of protein lysate was subjected to in-solution digestion method as previously outlined [49], with certain modifications. Briefly, samples were reduced using dithiothreitol, subsequently alkylated with chloroacetamide, and digested by Lys-C protease and trypsin. The resulting digested samples were subsequently acidified with trifluoroacetic acid (TFA) and purified using stop-and-go extraction (STAGE) tips [51], prior to mass spectrometry analysis. Statistical analysis and identification of differentially regulated proteins were conducted using DESeq2 (version 1.46.0) [52]. Lists of all significantly differentially regulated proteins are provided in the Supplementary Table 6. The pathway enrichment of the proteins was generated using the ReactomePA (Reactome Pathway Analysis) package from Bioconductor in R Studio [53] The pathway annotation networks were created with the ClueGo plug-in (version 2.5.10) in Cytoscape (version 3.10.3) [54].

### Quantification and statistical analysis

No statistical methods were used to predetermine sample size. Sample sizes were based on standard practice for in vitro molecular biology experiments. All experiments were performed with at least three independent biological replicates. Where applicable, experiments were conducted across multiple cell models, including three established meningioma cell lines and six patient-derived primary meningioma cultures, to ensure reproducibility and generalisability of the findings. Samples were not randomised, as experiments were conducted under controlled in vitro conditions. The investigators were not blinded to group allocation during experiments or outcome assessment. ImageJ software was used for immunoblotting densitometry and fluorescence image analysis of γ-H2AX foci and spheroids. Flow cytometry data were analysed using FlowJo software. Statistical analysis was performed with GraphPad Prism 9, employing a Student’s *t* test for two groups and one-way ANOVA (Dunn’s or Tukey) for three or more groups with normally distributed data. Non-parametric tests, including Mann-Whitney and Kruskal-Wallis, were used for non-normally distributed data. Data are presented as mean ± SEM.

## Supplementary information

Supplementary Figures

Supplementary Tables 1, 2, 3, 4, 7

Supplementary Table 5_Transcriptomics_List of significant DEGs

Supplementary Table 6_Proteomics_List of significantly regulated proteins

Original uncropped western blots

## Data Availability

The data that support the findings of this study are available from the corresponding author upon reasonable request.
