# Supplementary material for: Targeting HDACs with dacinostat amplifies radiation-induced DNA damage via sustained phosphorylated p53 and repair inhibition in aggressive meningioma
Source: Cell Death Dis. 2026 Jun 2;17(1):688. doi: 10.1038/s41419-026-08899-4 (PMC13442888; doi:10.1038/s41419-026-08899-4)
Supplement: Supplementary file 1 [file 41419_2026_8899_MOESM1_ESM.pdf]

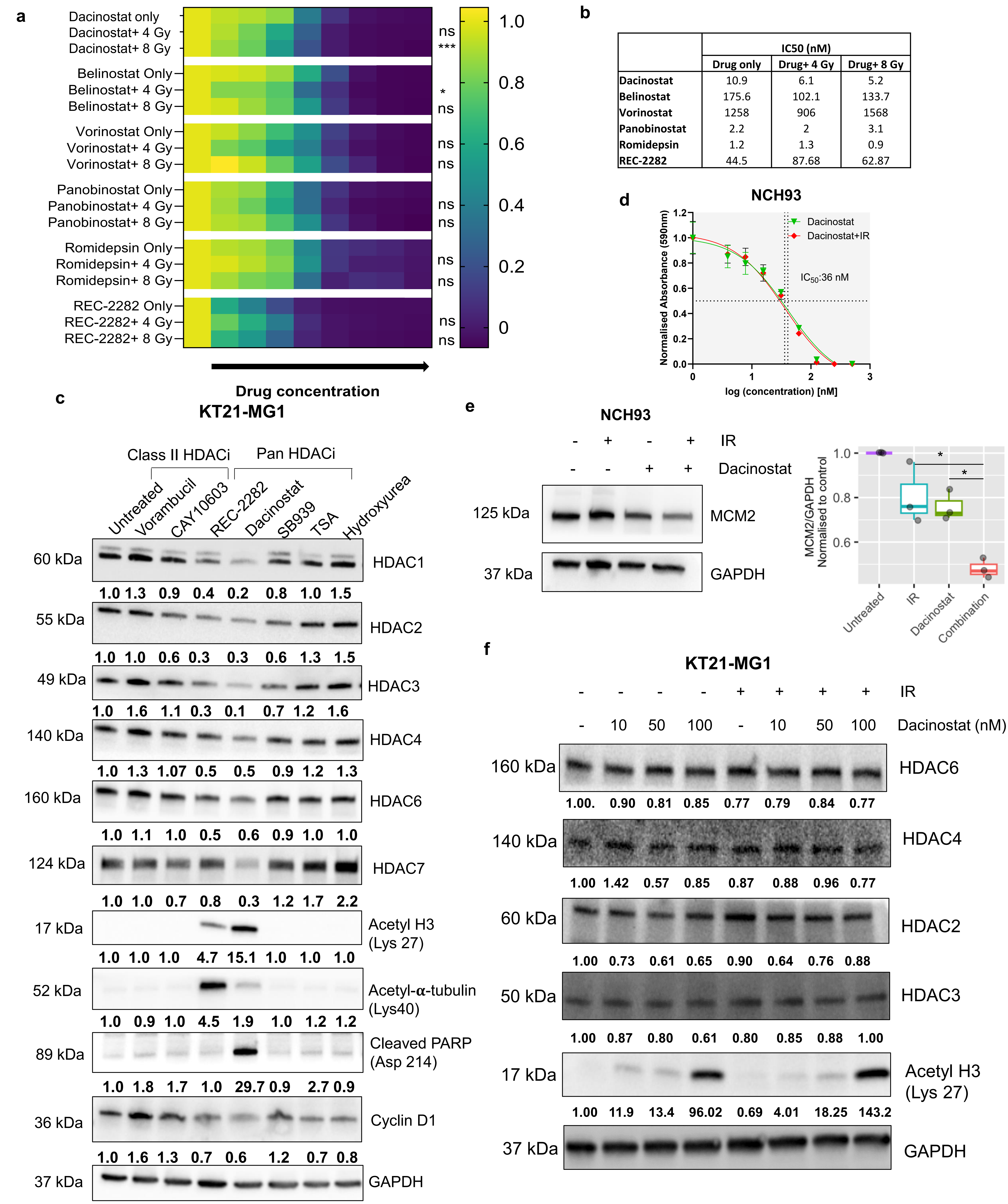

	IC50 (nM)		
	Drug only	Drug+ 4 Gy	Drug+ 8 Gy
Dacinostat	10.9	6.1	5.2
Belinostat	175.6	102.1	133.7
Vorinostat	1258	906	1568
Panobinostat	2.2	2	3.1
Romidepsin	1.2	1.3	0.9
REC-2282	44.5	87.68	62.87

Supplementary Figure S1. Dacinostat exhibited superior efficacy compared to other HDAC inhibitors. (a) Heatmap showing the effects of combination treatment with Dacinostat, Belinostat, Vorinostat, Panobinostat, and REC-2282 together with radiotherapy. Dacinostat and Belinostat demonstrated significant effects in combination. (b) Corresponding IC50 values for the treatments shown in (a). (c) A comparative analysis of dacinostat with other HDAC inhibitors at a concentration of 100 nM revealed a notable reduction in the expression of HDAC proteins, alongside an increase in acetylation and expression of cleaved PARP. (d) The viability assay demonstrated that, although dacinostat administered alone reduced the viability of NCH93, no significant difference was observed between the effects of dacinostat alone and its combination with IR (4 Gy). (e) Protein expression in NCH93 indicates that the expression of MCM2 was synergistically reduced following the combination treatment. (f) Protein expression data indicate that a low dose of dacinostat exerts a diminished effect on HDAC expression and acetylation in comparison to a higher dosage.

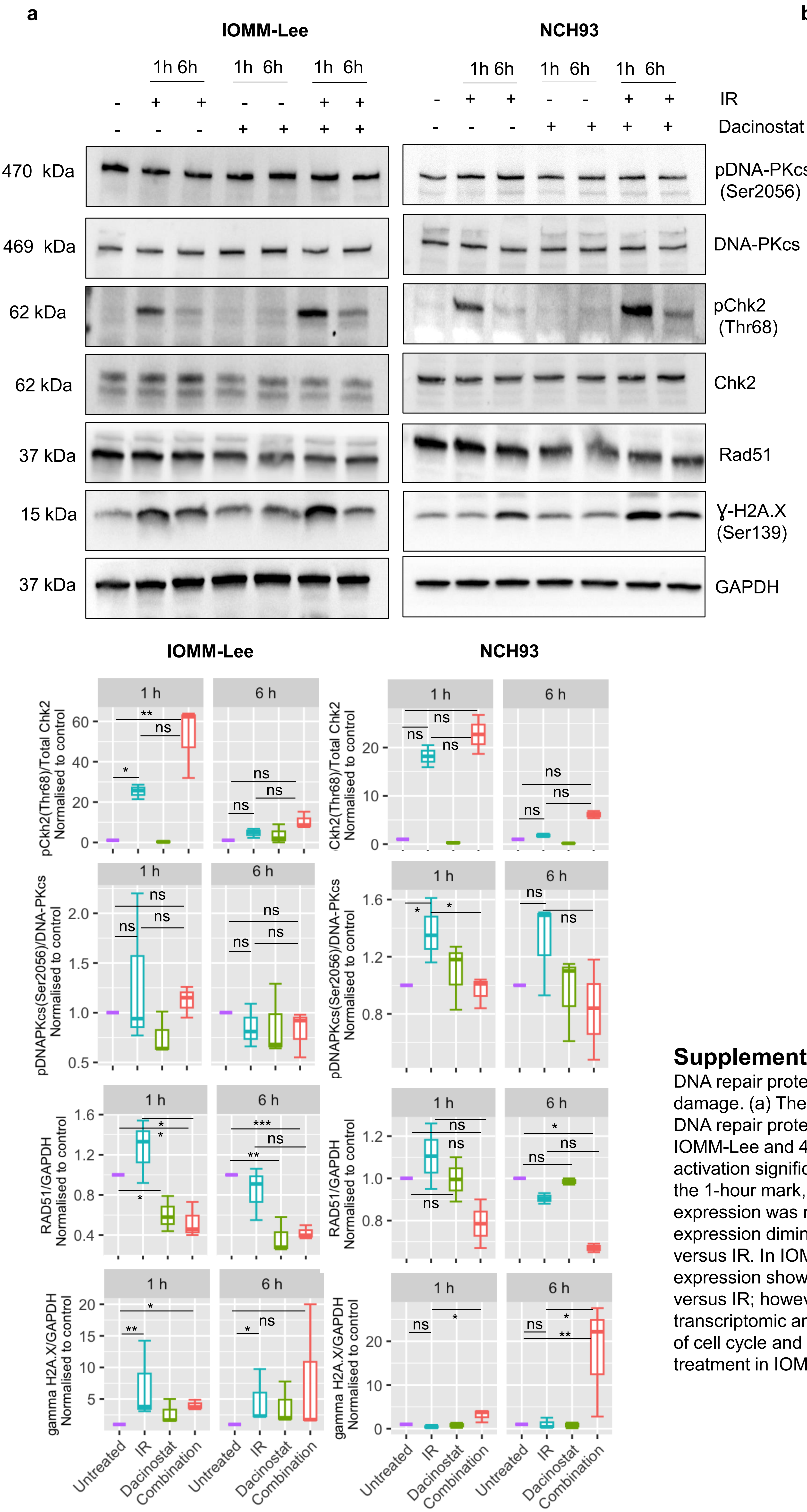

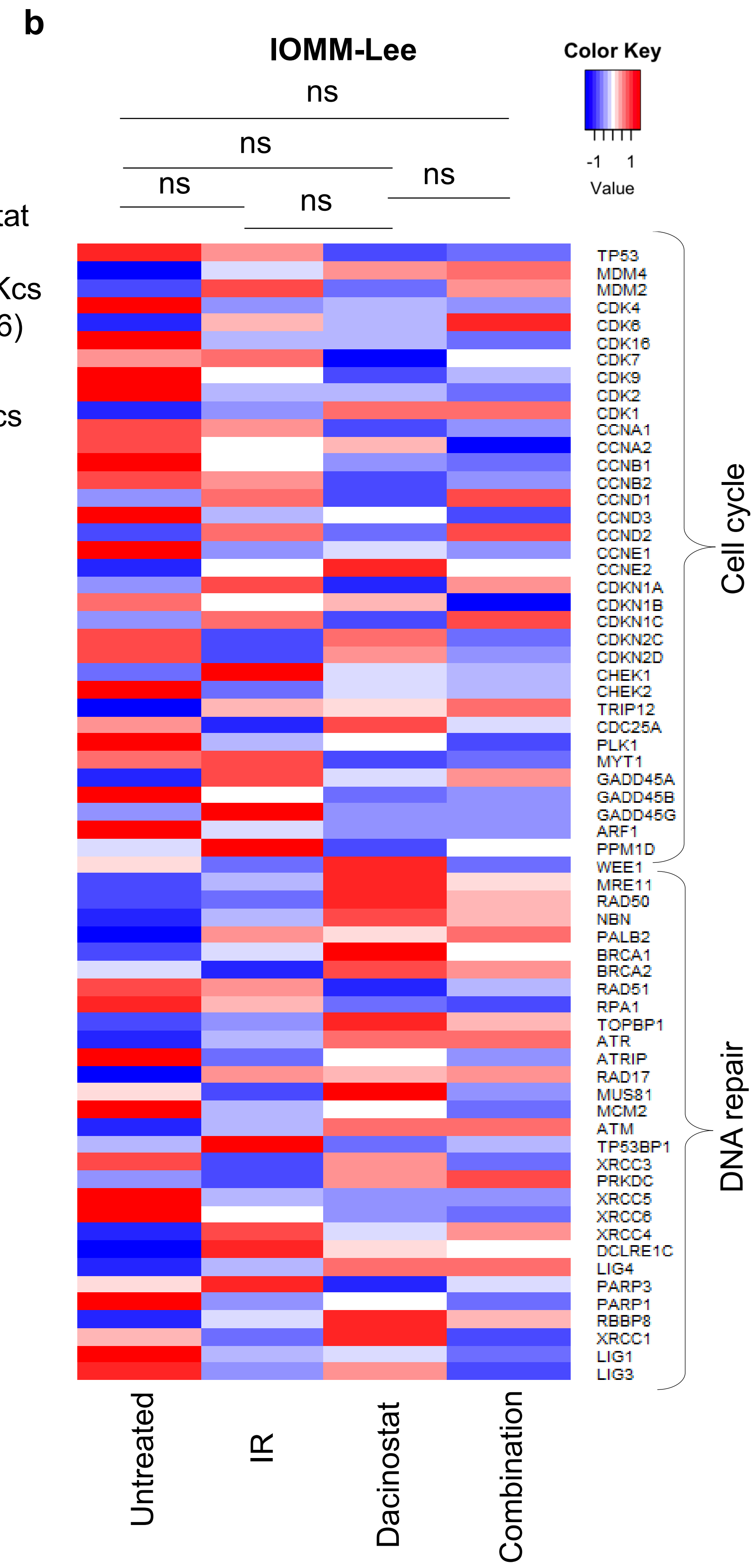

Supplementary Figure S2. Dacinostat with IR reduced DNA repair protein expression and enhanced IR-induced DNA damage. (a) The western blot showed modulation of cell cycle and DNA repair protein expression at 1- and 6-hours post-IR (2 Gy for IOMM-Lee and 4 Gy for NCH93). Analysis showed that Chk2 activation significantly increased after the combination treatment at the 1-hour mark, but it gradually declined. Prolonged gH2A.X expression was noted, while DNA-Pkcs activation and RAD51 expression diminished in NCH93 with the combination treatment versus IR. In IOMM-Lee, DNA-Pkcs activation and gH2A.X expression showed no changes with the combination treatment versus IR; however, dacinostat reduced Rad51 expression. (b) The transcriptomic analysis heatmap indicated no substantial regulation of cell cycle and DNA repair genes following the combination treatment in IOMM-Lee.

Supplementary Figure S2

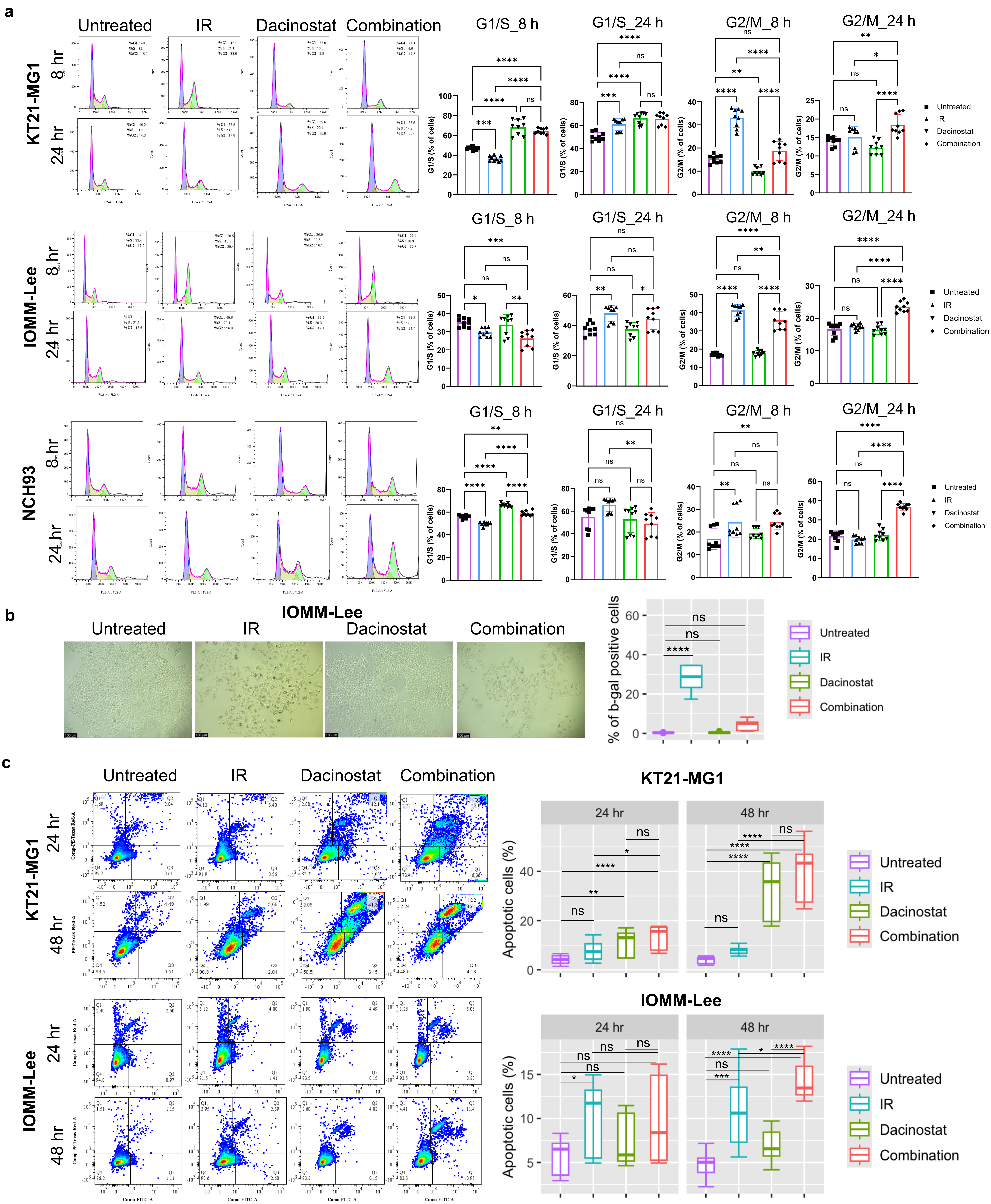

Supplementary Figure S3. Pretreatment with dacinostat prior to radiation enhanced cell arrest and cell death. (a) Cell cycle analysis shows that G1/S arrest is predominantly seen in KT21-MG1, while IOMM-Lee and NCH93 exhibit a significantly increased G2/M arrest at 24 hours post-IR (2 Gy for IOMM-Lee, and 4 Gy for KT21-MG1 and NCH93). (b) β-galactosidase staining 4 days post-IR (2 Gy) showed increased cellular senescence in radiation-alone exposed cells; this was not observed in cells subjected to combination treatment. (c) The time-point study utilising Annexin-V/PI flow cytometry demonstrated that cells pretreated with dacinostat before IR (2 Gy for IOMM-Lee and 4 Gy for KT21-MG1) exhibited a gradual increase in apoptosis.

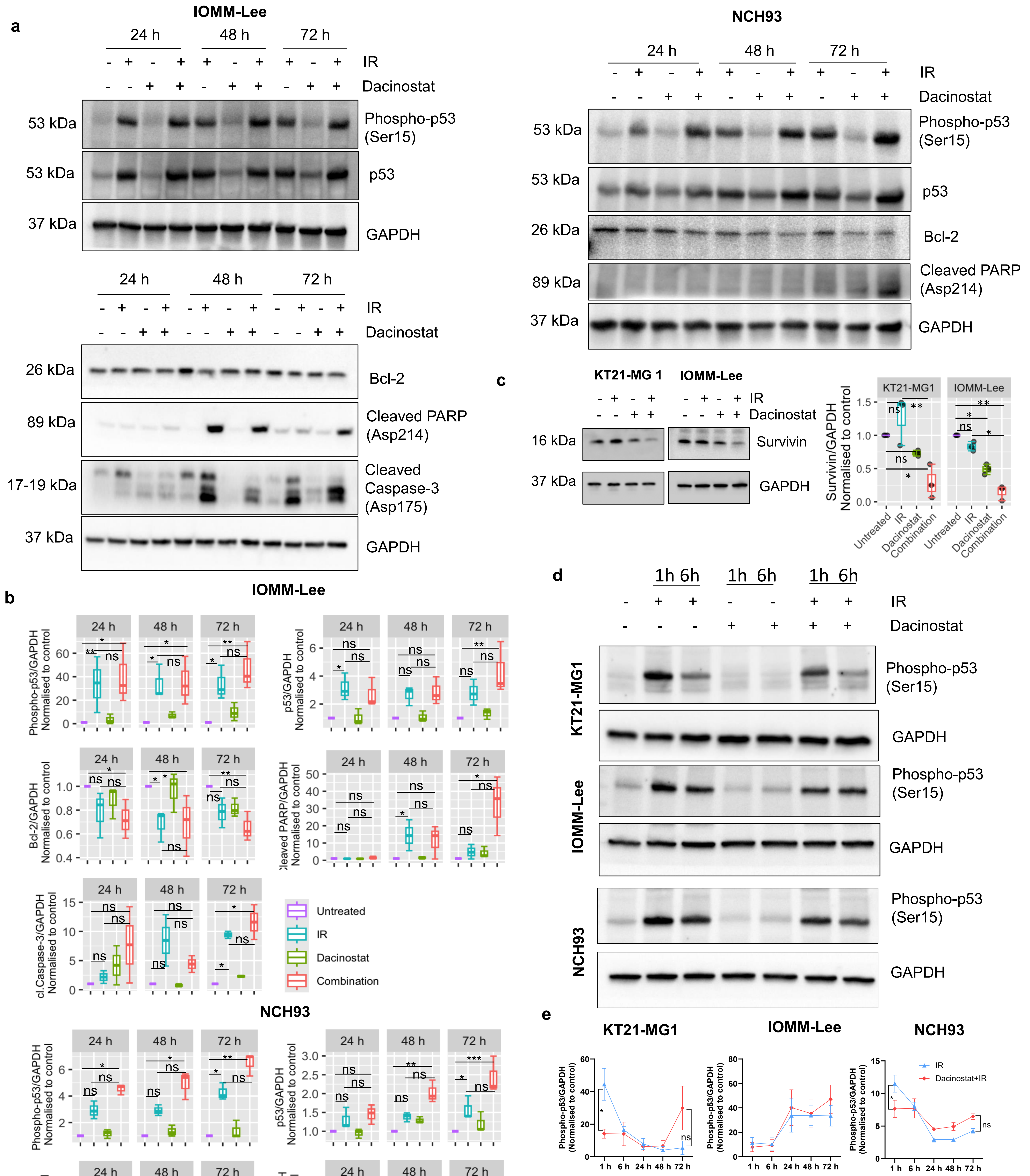

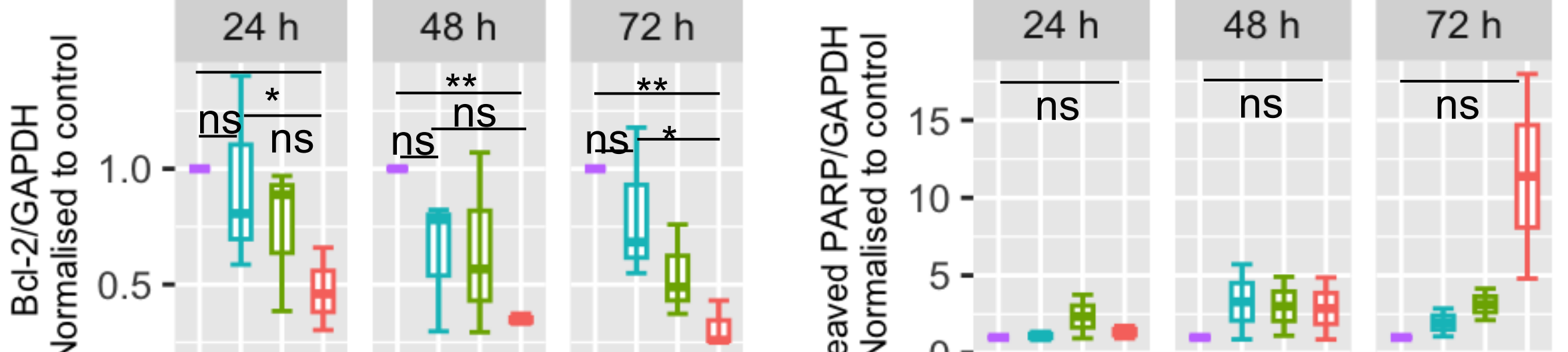

Supplementary Figure S4. The combination of dacinostat and IR prolonged the expression of phospho-p53 (Ser15) compared to IR alone. (a) An illustrative image of the western blot displays the detection of phospho-p53 (Ser15), total p53, and apoptosis indicators at various time points post-IR (2 Gy for IOMM-Lee and 4 Gy for NCH93). (b) Protein quantification shows that cells with the combination treatment sustained high phospho-p53 (Ser15) and p53 levels, peaking at 72 hours post-IR, which aligns with maximum apoptosis. (c) Representative western blot image for the expression of survivin, and the analysis revealed the synergistic reduction of survivin following the combination treatment. (d) An illustrative image of the wester (d) An illustrative image of the western blot displays the detection of phospho-p53 (Ser15) and total p53 at 1- and 6-hours post-IR (2 Gy for IOMM-Lee and, 4 Gy for KT21-MG1 and NCH93). (e) A graphical representation of phospho-p53 (Ser15) dynamics across various time intervals following IR and combination treatment.

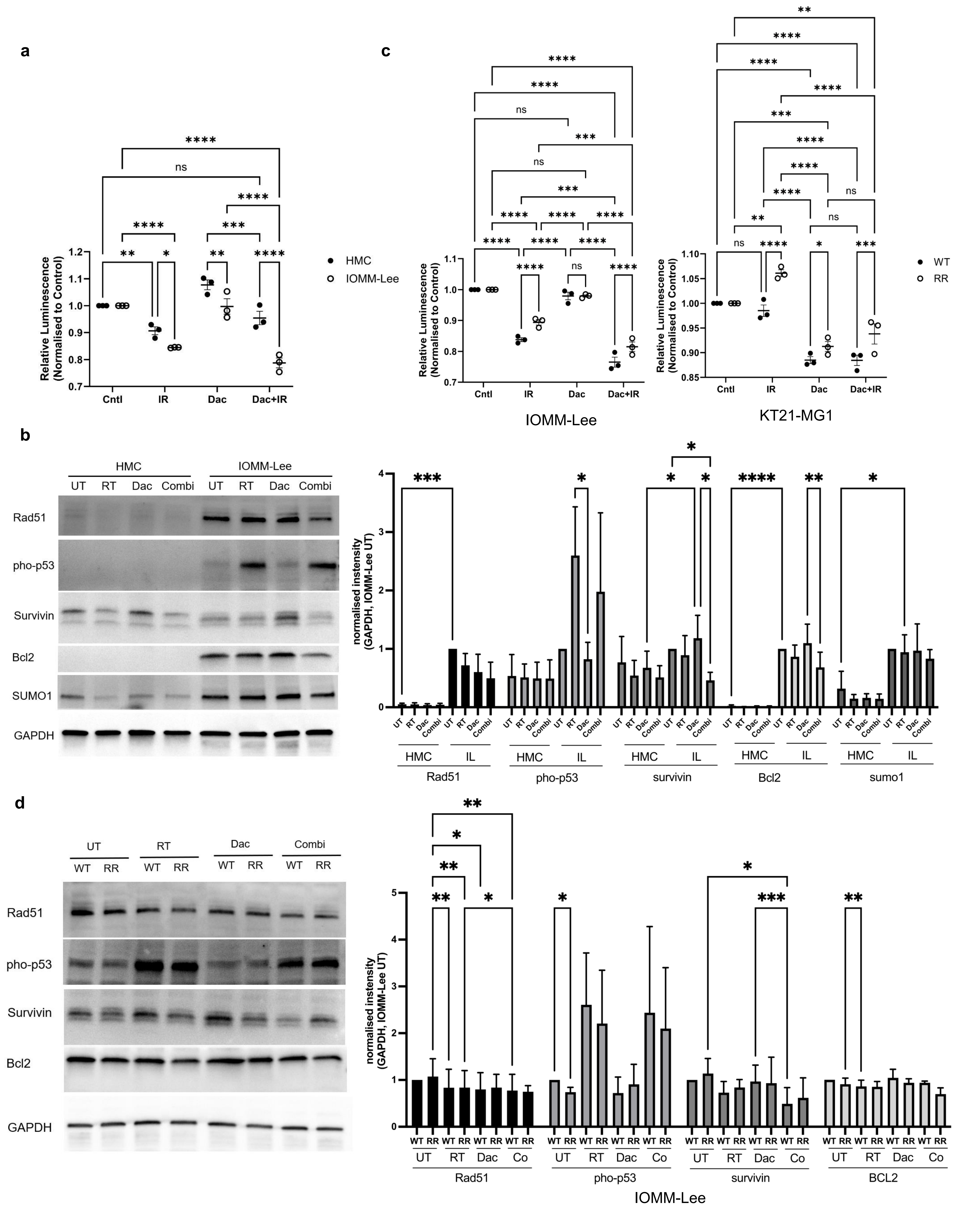

Supplementary Figure S5. (a) Cell viability assays in HMC and IOMM-Lee cells treated with ionising radiation (IR, 2 Gy), Dacinostat (11 nM), or the combination. Dacinostat was applied 24 hr prior to IR, and viability was measured 48 hr post-radiation. (b) Western blot analysis 24 hr after IR showing Rad51, phospho-p53, Survivin, Bcl2, and SUMO1 expression under the same treatment conditions as (a). (c) Cell viability assays in IOMM-Lee and KT21-MG1 cells under IR (2 Gy for IOMM-Lee; 4 Gy for KT21-MG1) and Dacinostat (11 nM for IOMM-Lee; 36 nM for KT21-MG1) alone or in combination. Dacinostat was applied 24 hr prior to IR, and viability was measured 48 hr (IOMM-Lee) or 96 hr (KT21-MG1) post-radiation. Radioresistant cells were generated by repeated IR exposure (IOMM-Lee: 2 Gy × 5 every two weeks; KT21-MG1: 4 Gy × 5 every two weeks). (d) Western blot analysis of Rad51, phospho-p53, Survivin, and Bcl2 in wild-type and radioresistant IOMM-Lee cells under the same treatment conditions.

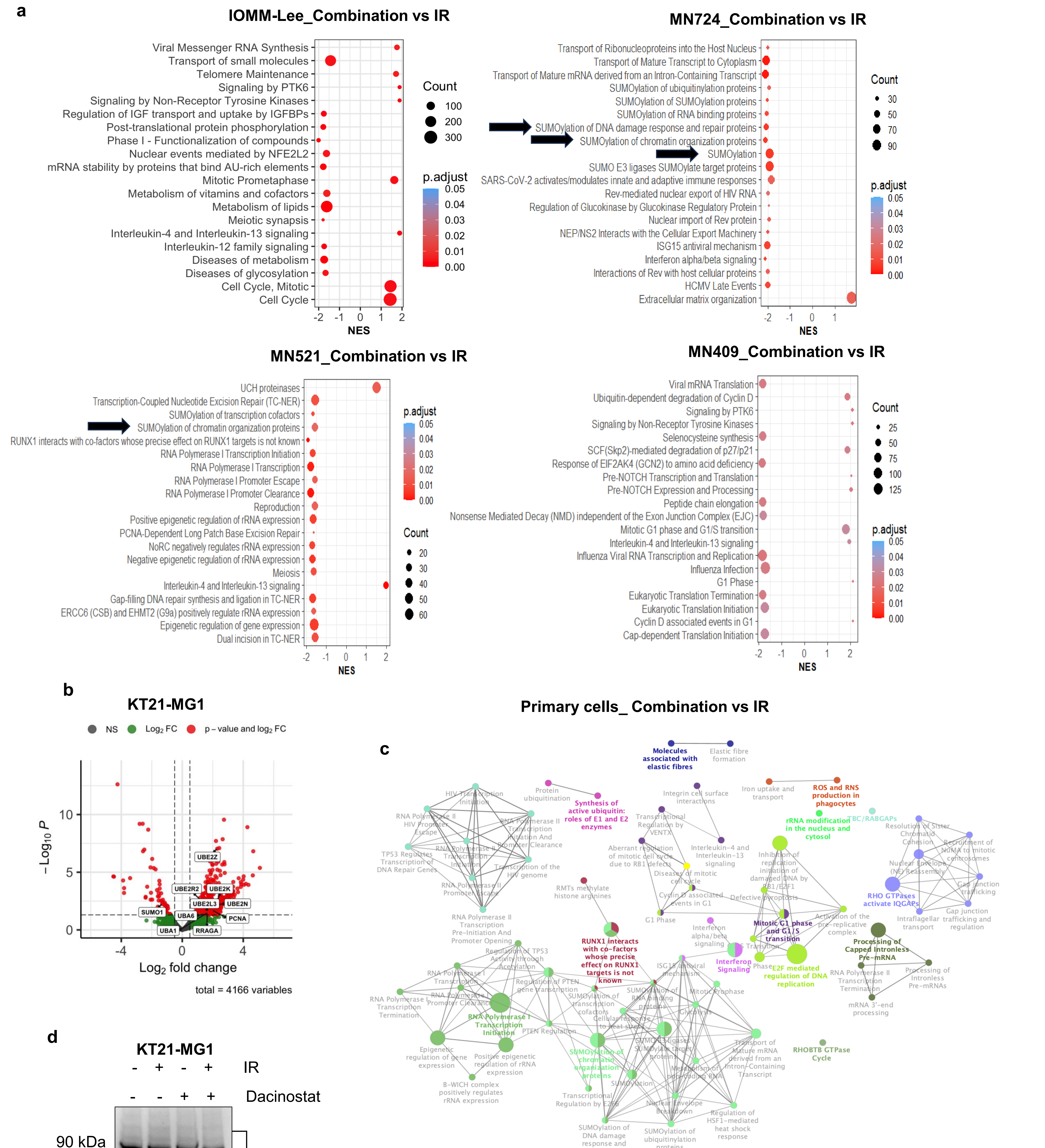

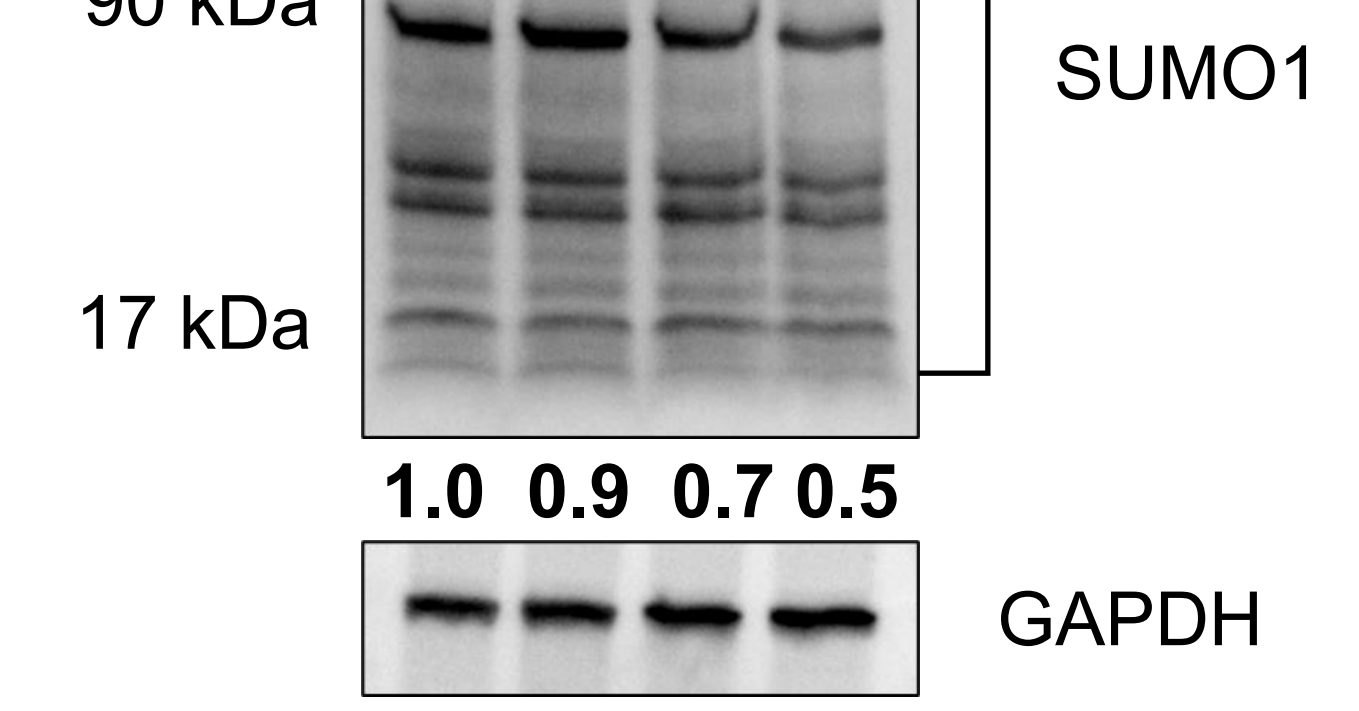

Supplementary Figure S6. SUMOylation and its related pathways are downregulated following the combination treatment. (a) The dot plot generated from ReactomePA, which compares the effects of combination treatment and IR (2 Gy for IOMM-Lee and 12 Gy for primary cells), illustrates the top 20 enriched pathways in IOMM-Lee and three primary cell types. The colour of the dots denotes the significance of expression change (adjusted p-value), whereas the size reflects the number of enriched proteins. (b) The volcano plot of DEGs from the DEseq2 analysis of KT21-MG1 indicated that the SUMO1 protein was significantly downregulated, whereas proteins associated with ubiquitination were upregulated. (c) The results derived from the DEseq2 analysis of primary cells, in conjunction with the outcomes obtained from ReactomePA via the ClueGO/Cluepedia plug-in, were visualised in Cytoscape. This visualisation demonstrated that SUMOylation and its associated pathways are regulated after the combination treatment. Terms that are grouped with a kappa score of ≥ 0.4 are represented as nodes in various colours. (d) Western blot validation of the SUMO1 protein in KT21-MG1 revealed a synergistic decrease in SUMO1 following combination treatment.

a

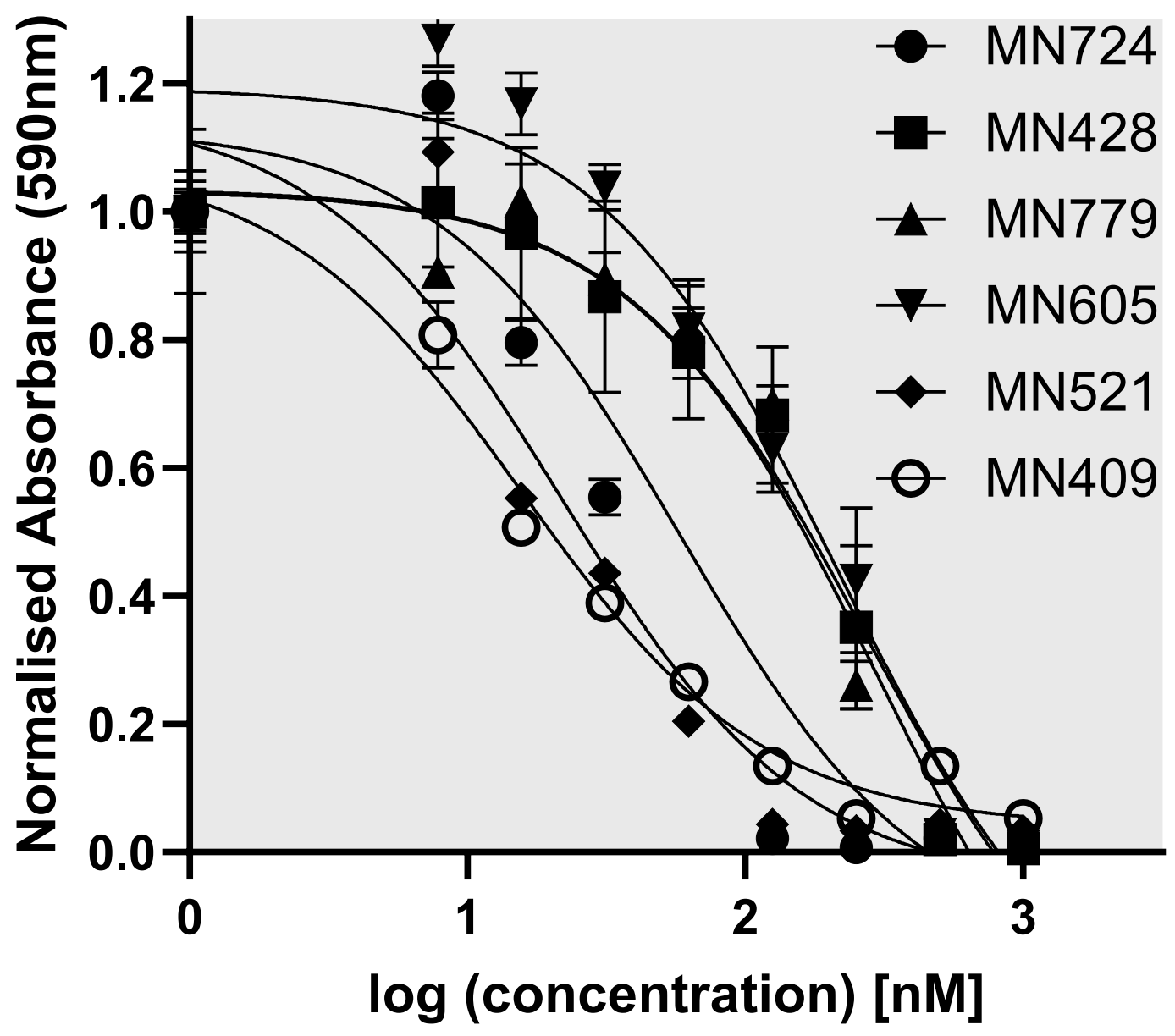

Primary cells	IC50 (nM)	95% CI
MN724	142	70.60 to 299.4
MN428	190	113.1 to 373.6
MN779	175	94.36 to 326.0
MN605	150	88.63 to 508.9
MN521	72	37.90 to 140.4
MN409	54	27.25 to 108.1

b

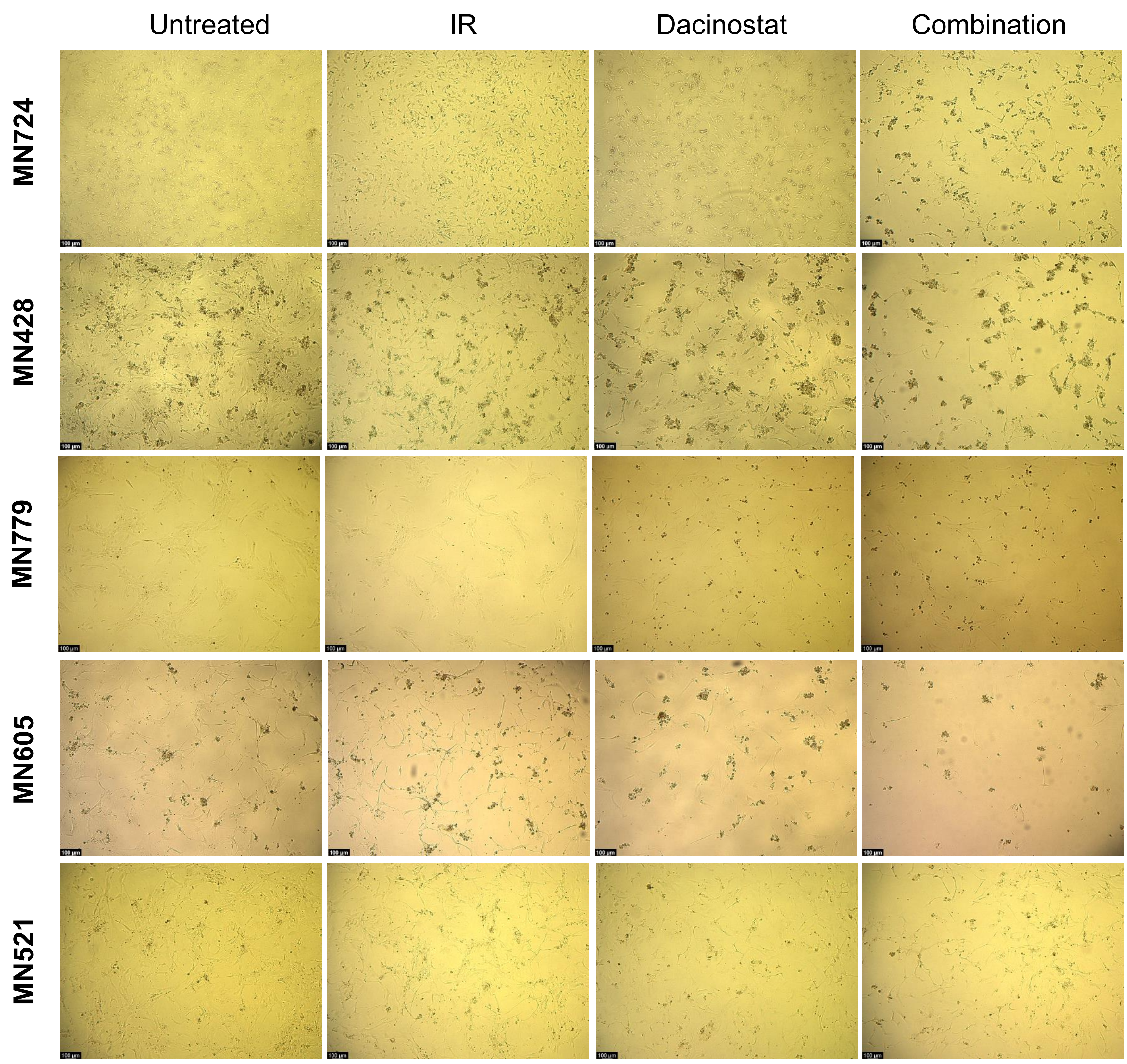

Supplementary Figure S7. Primary cells exhibited varying sensitivity to dacinostat treatment. (a)The viability of primary cells diminished following treatment with various concentrations of dacinostat, and the corresponding IC50 values were derived from the graph. (b) Representative image of primary cells after treatment with IR (12 Gy), dacinostat, and both.

a

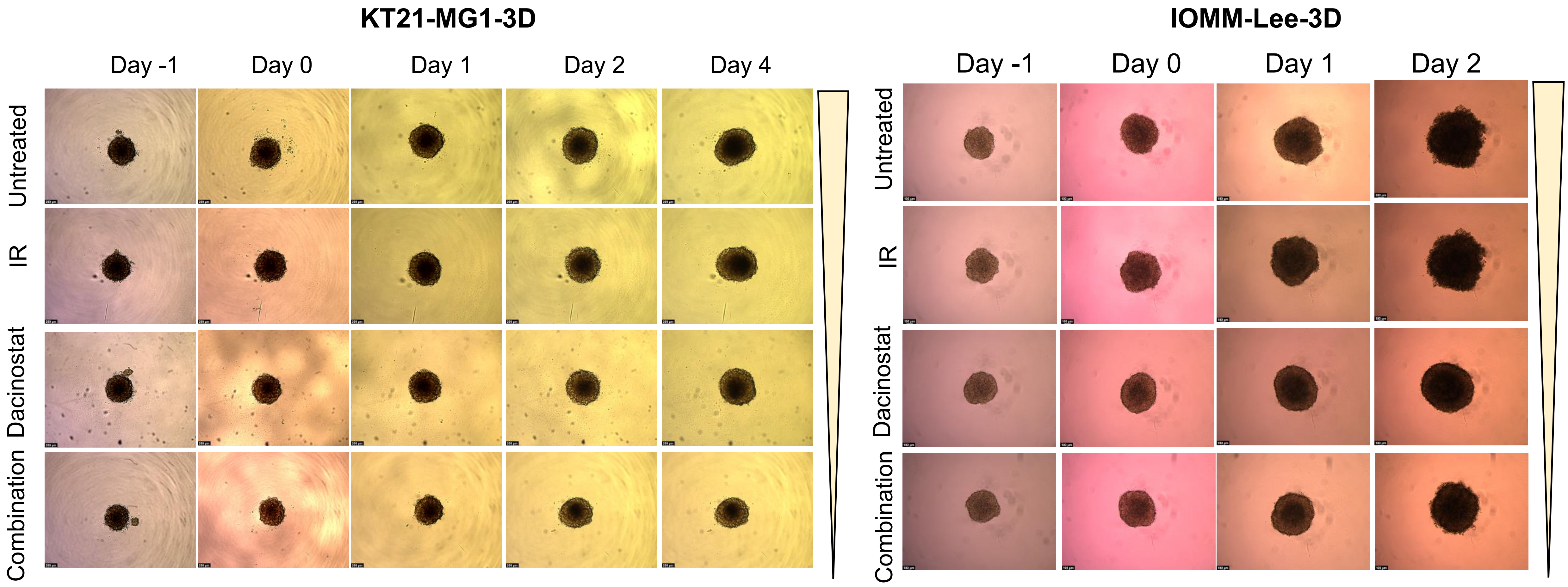

b

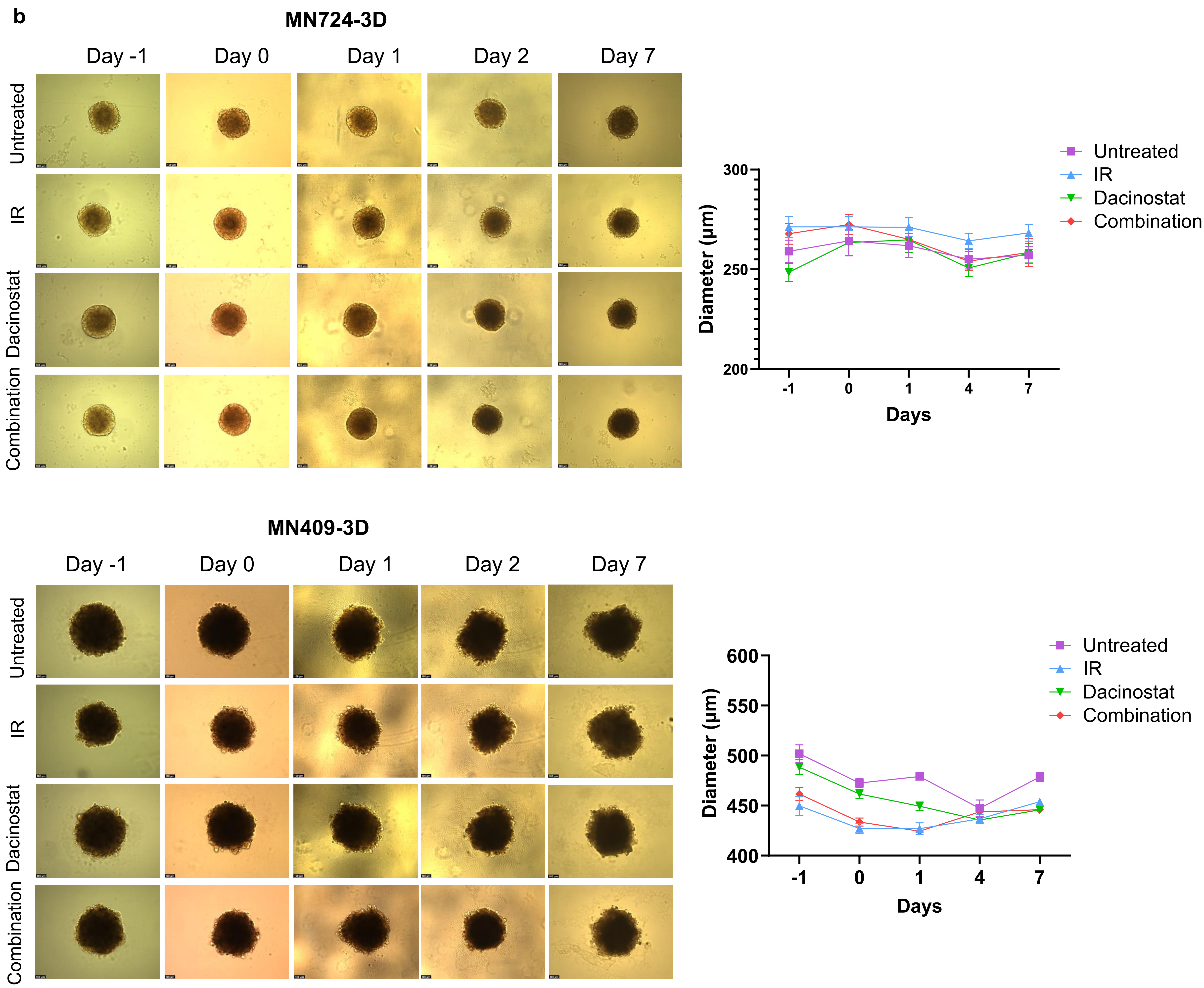

Supplementary Figure S8. Pretreatment with dacinostat reduced the growth of 3D spheroids derived from cells following IR. (a) An illustrative representation is presented, depicting the synergistic decrease in the growth of spheroids derived from immortalised cell lines following combination treatment. (b) An illustrative representation of the growth of spheroids derived from primary cells, along with the graphical analysis, demonstrates no significant difference in growth size following combination treatment, attributable to the limited proliferation potential of primary cells.
