# Supplementary material for: Targeting HDACs with dacinostat amplifies radiation-induced DNA damage via sustained phosphorylated p53 and repair inhibition in aggressive meningioma
Source: Cell Death Dis. 2026 Jun 2;17(1):688. doi: 10.1038/s41419-026-08899-4 (PMC13442888; doi:10.1038/s41419-026-08899-4)

Figure 1

d

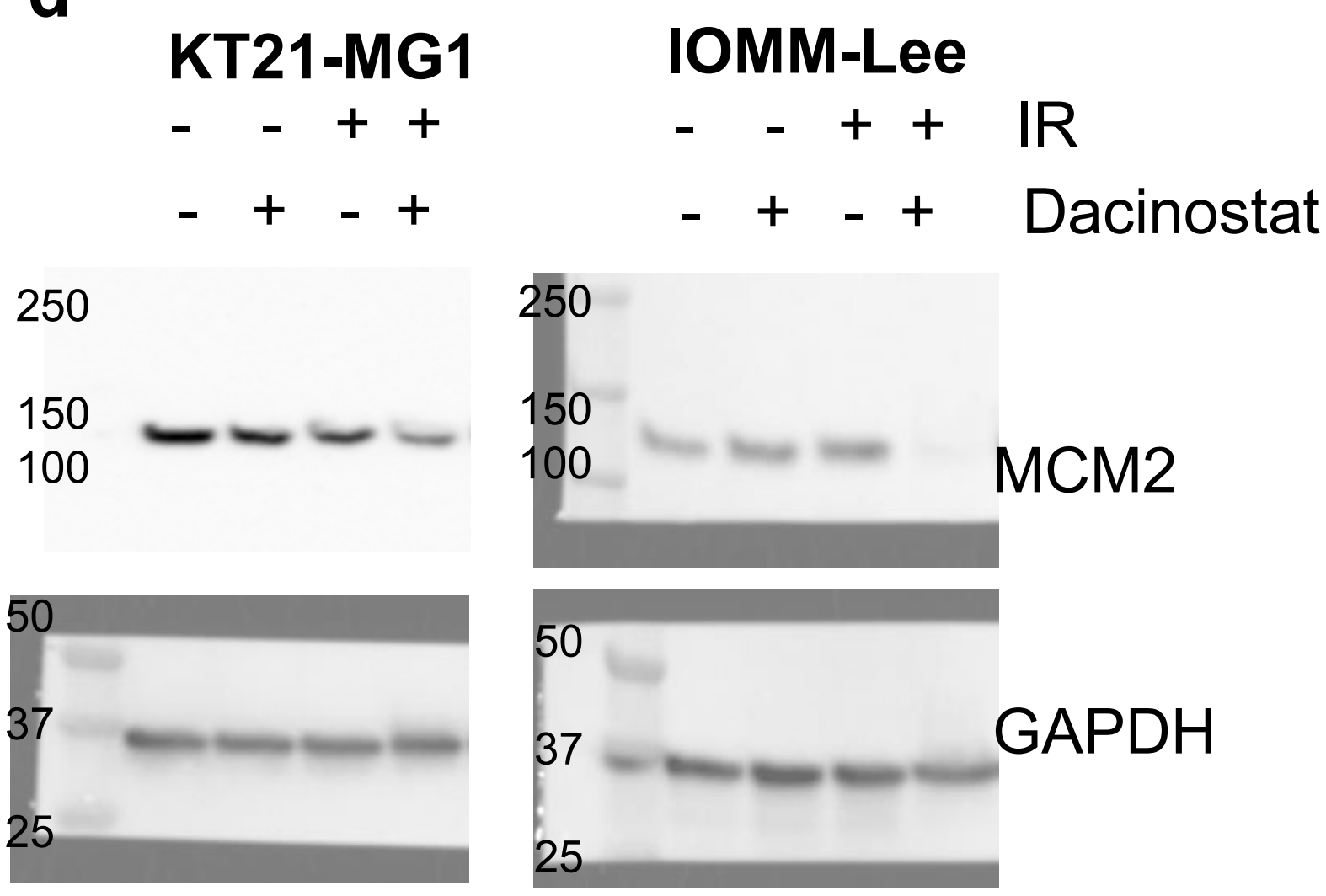

Figure 2

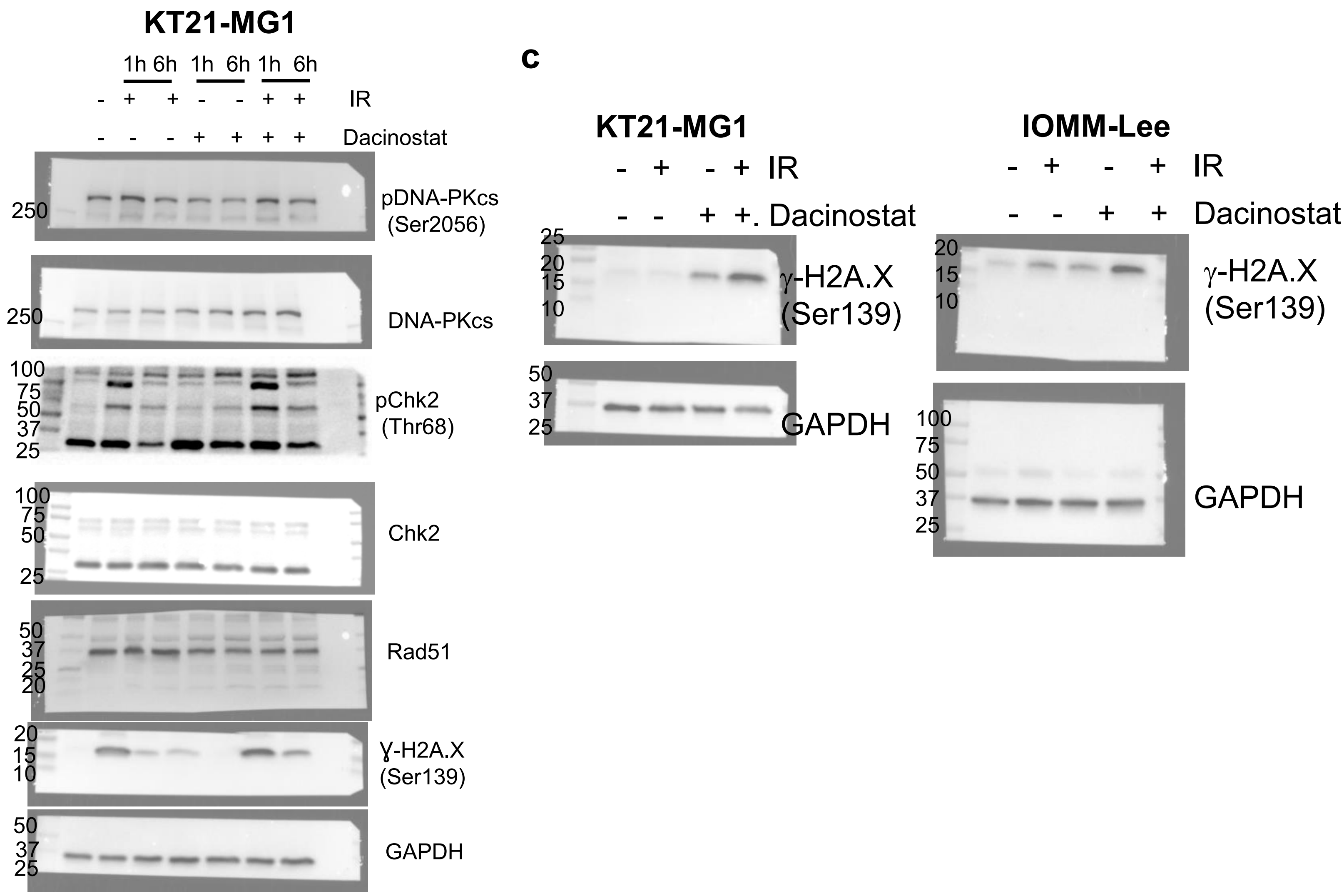

Figure 3

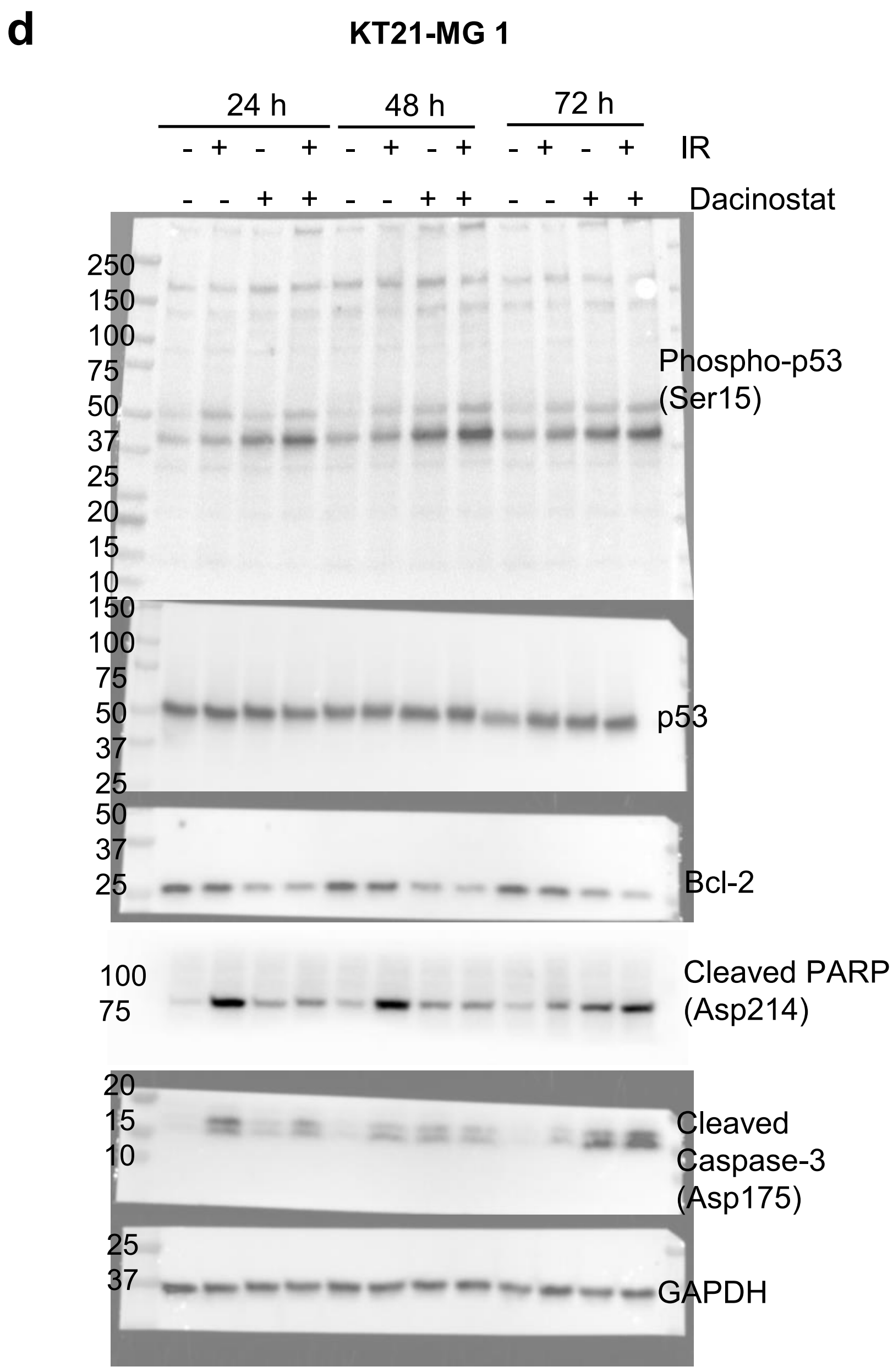
d
KT21-MG 1
24 h
48 h
72 h
- + - + - + - + - + - +
IR
- - + + - - + + - - + +
Dacinostat
250
150
100
75
50
37
25
20
15
10
Phospho-p53
(Ser15)
150
100
75
50
37
25
p53
50
37
25
Bcl-2
100
75
Cleaved PARP
(Asp214)
20
15
10
Cleaved
Caspase-3
(Asp175)
25
37
GAPDH

Figure 5

d

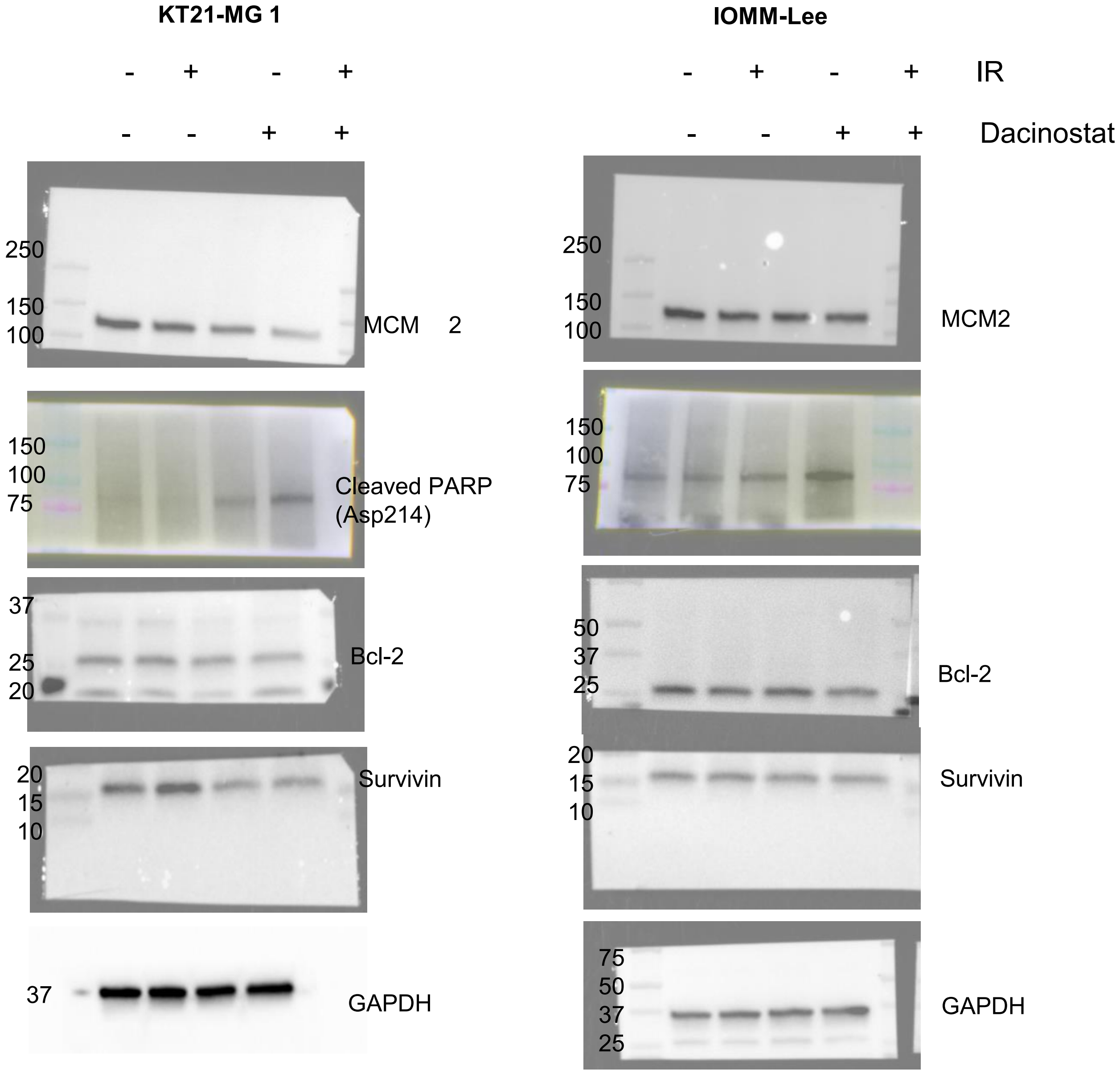

KT21-MG 1
IOMM-Lee
- + - + IR
- - + + Dacinostat
250
150
100
MCM 2
MCM2
150
100
75
Cleaved PARP (Asp214)
37
25
20
Bcl-2
50
37
25
20
15
10
Survivin
37
GAPDH
75
50
37
25

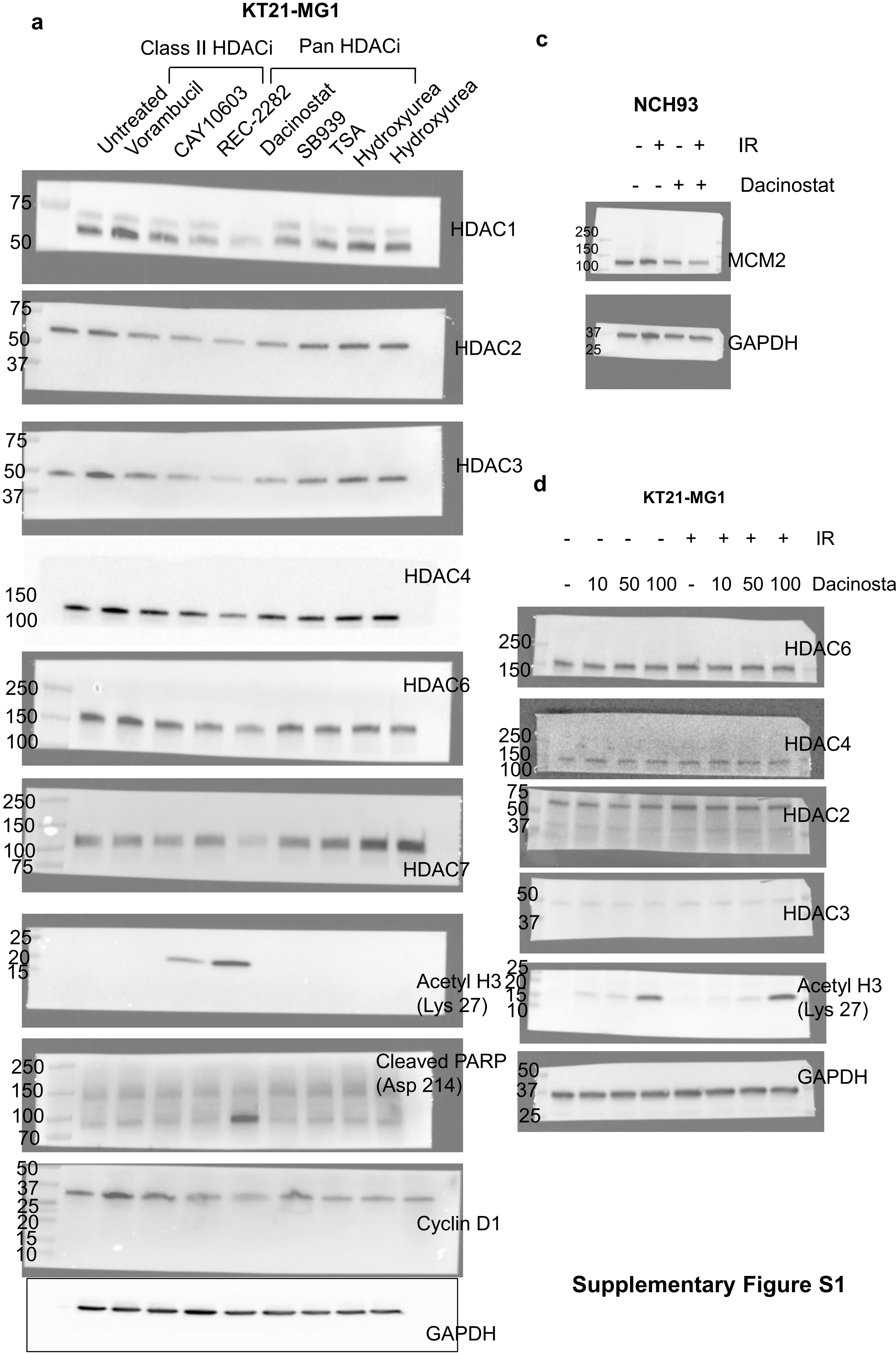

Supplementary Figure S1

Supplementary Figure S2

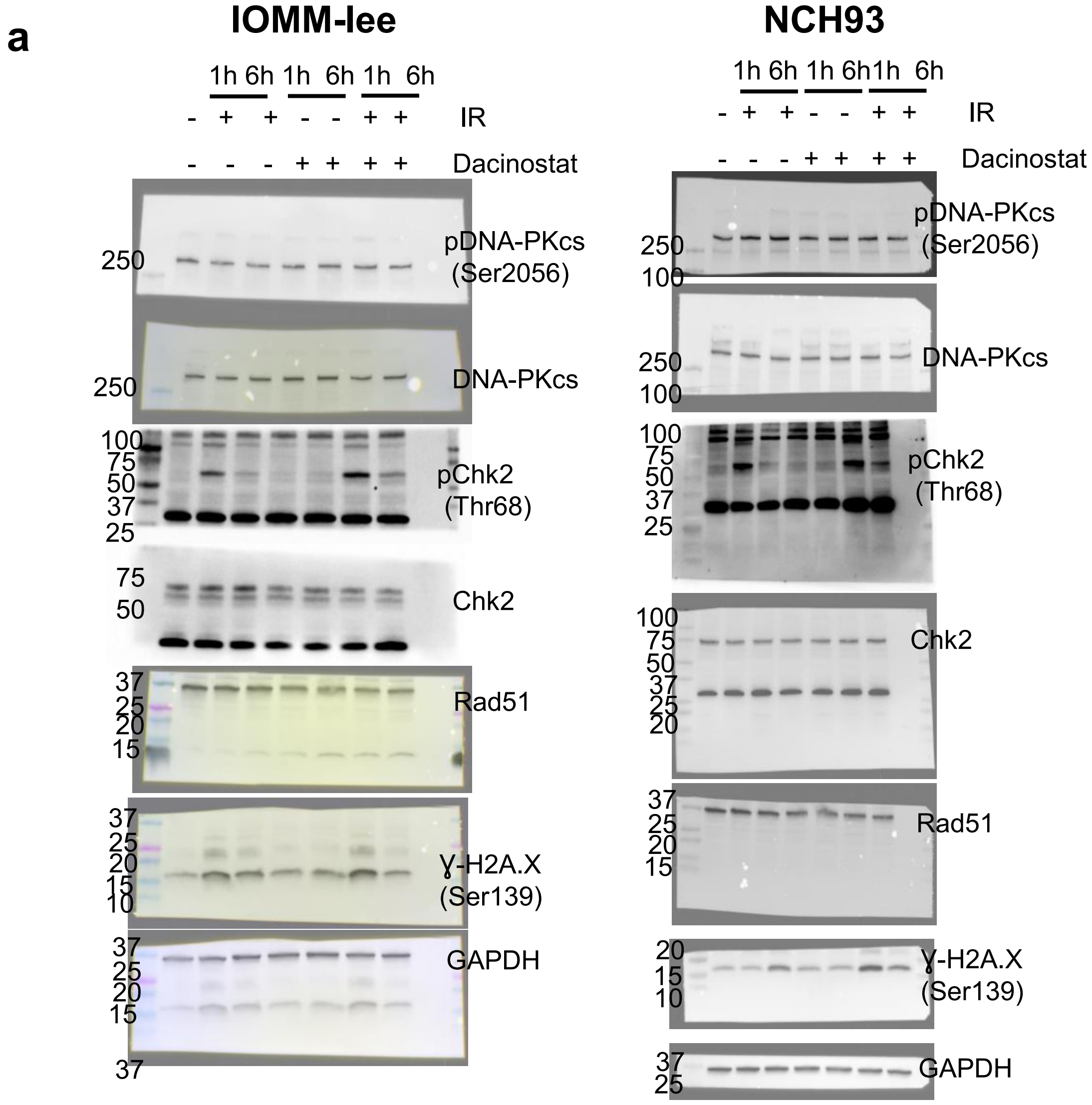

a

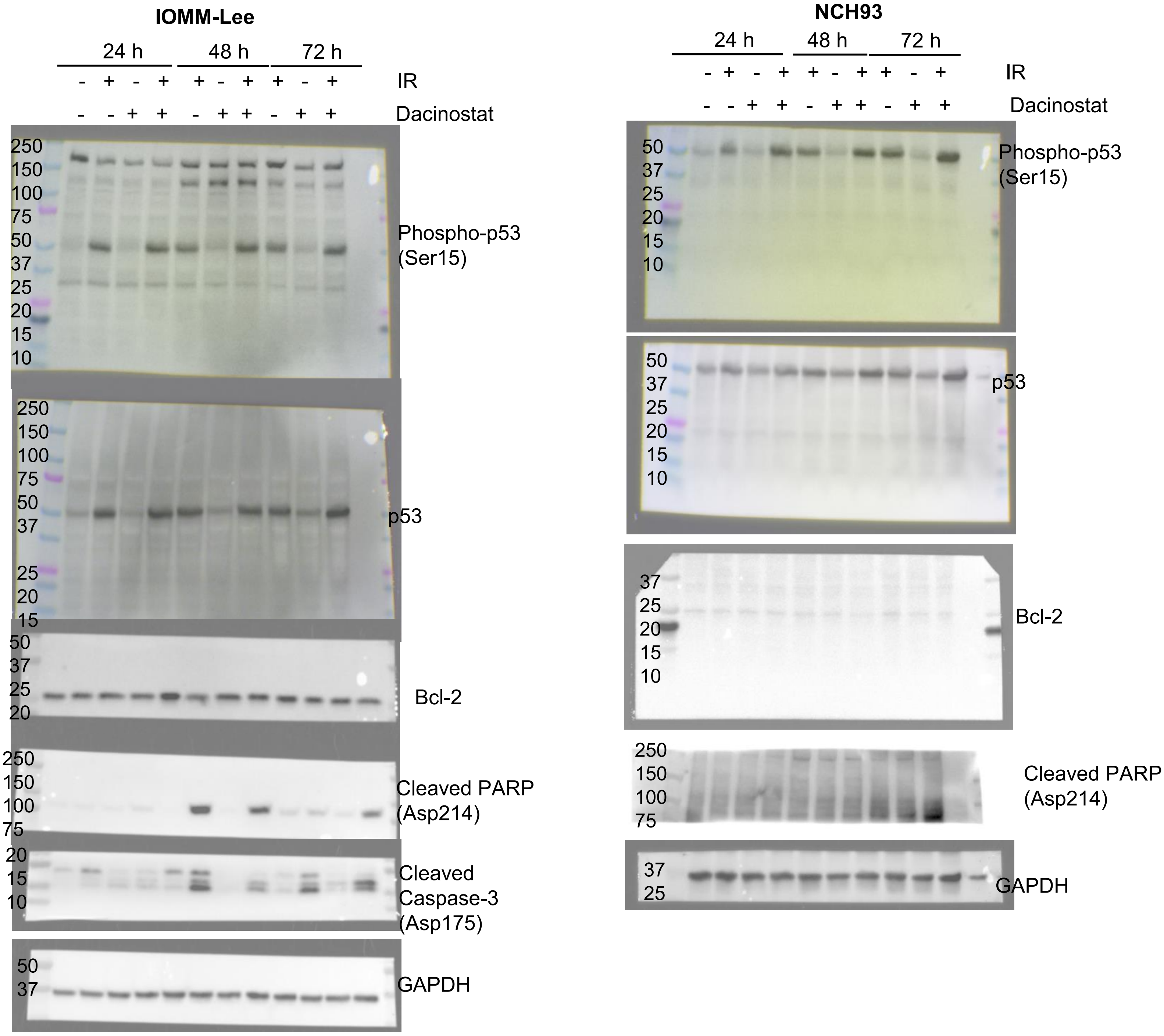

d

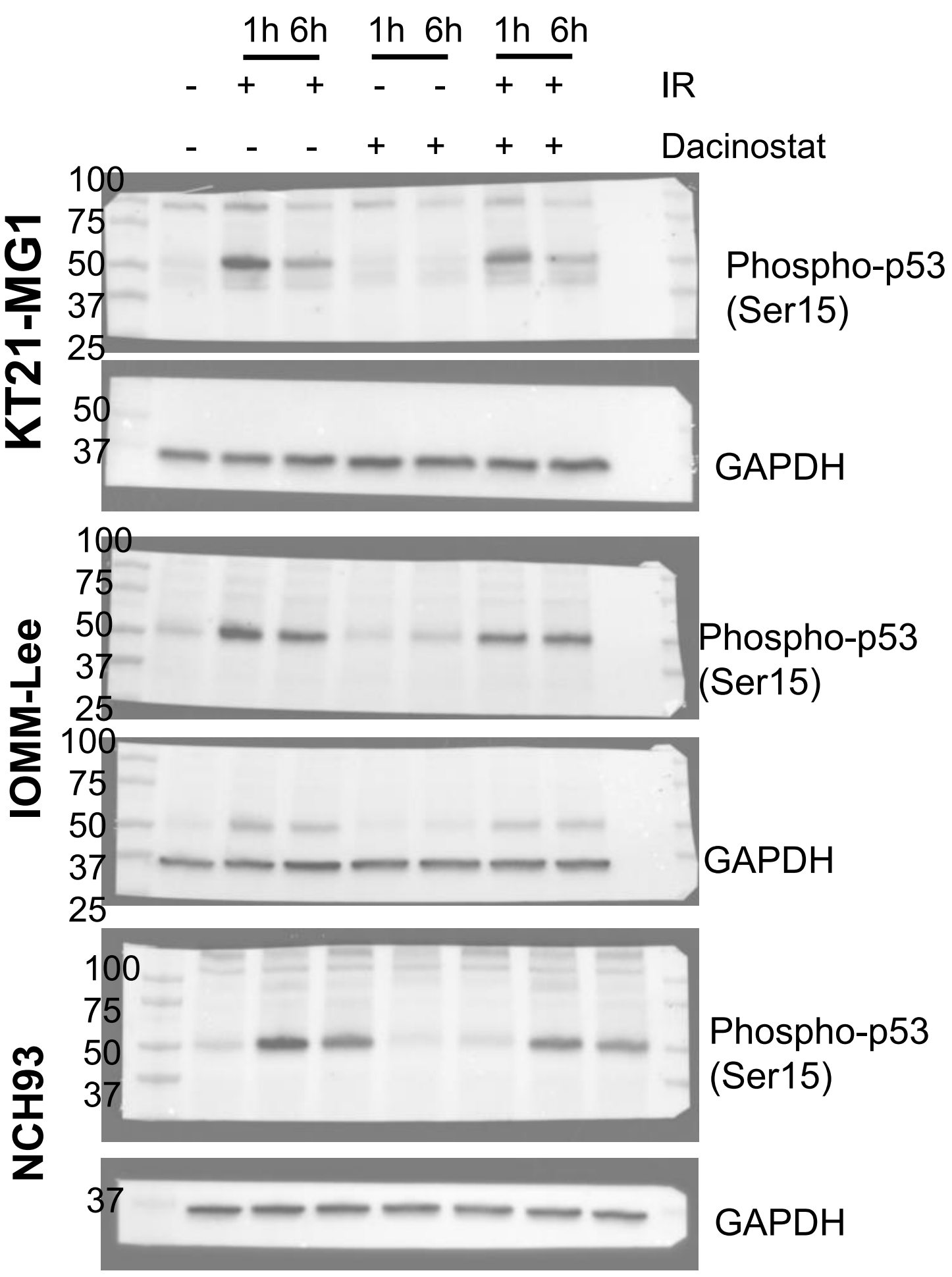

Supplementary Figure S4

Supplementary Figure S5

b

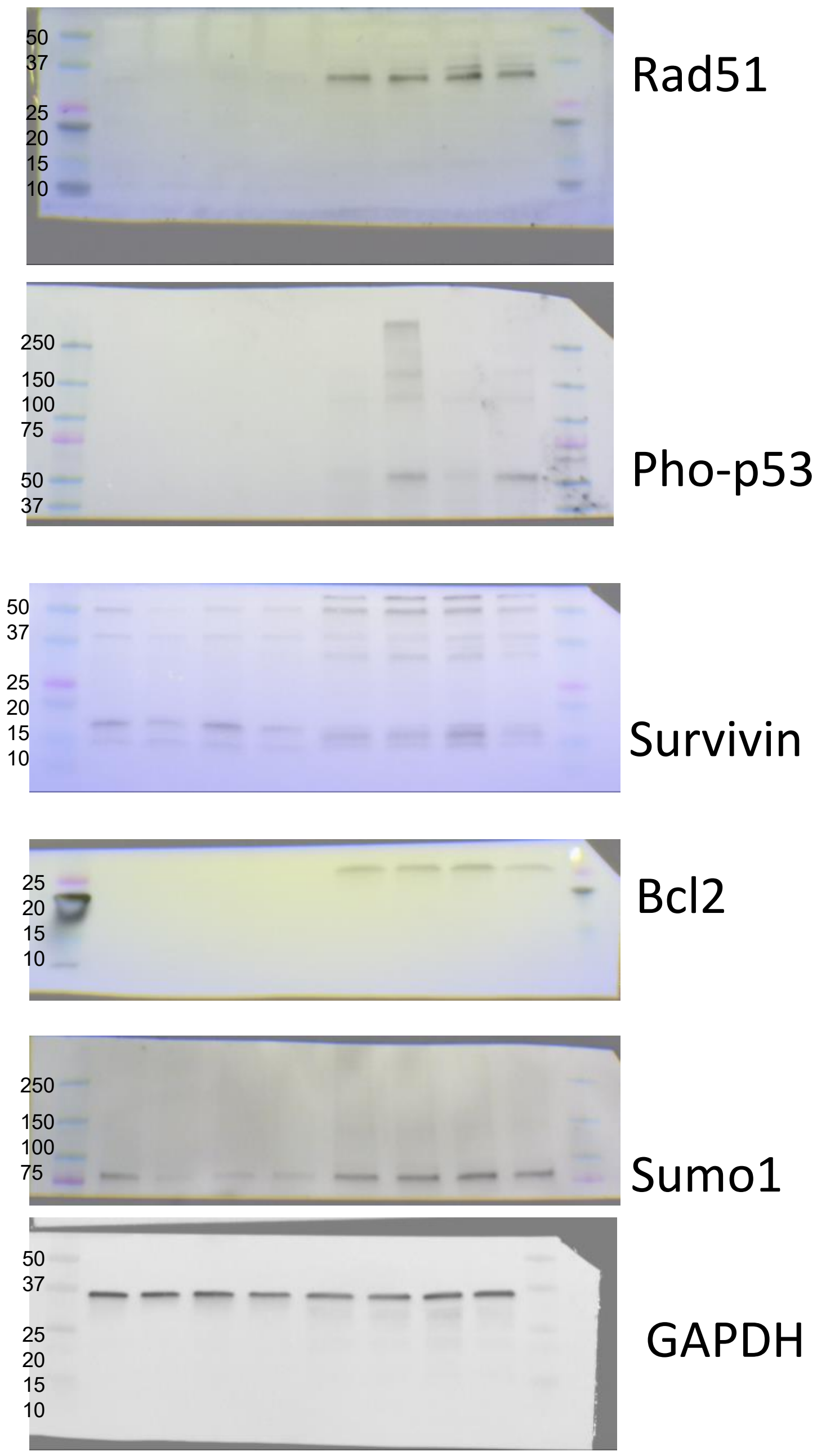

d

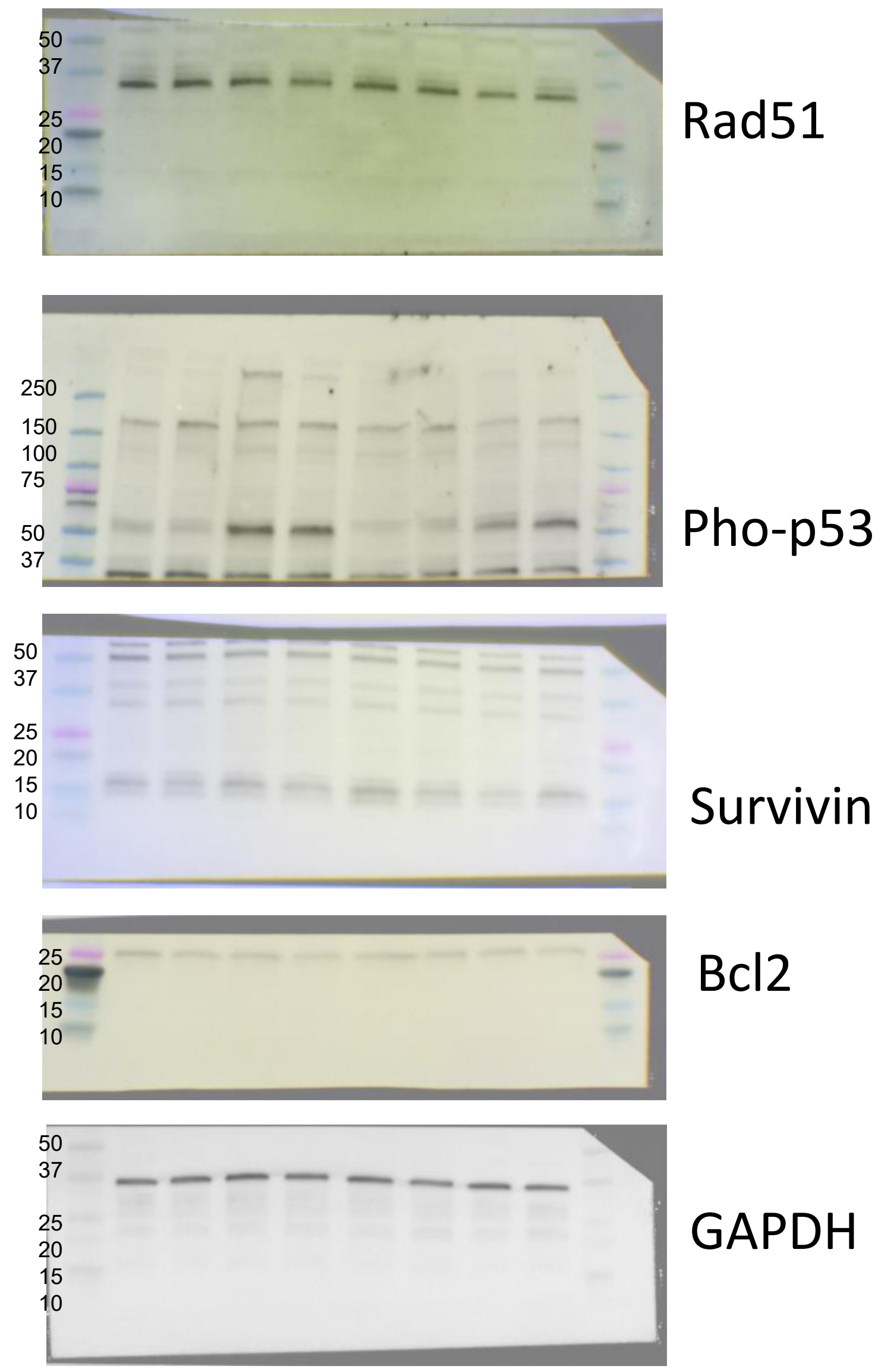

Supplementary Figure S6

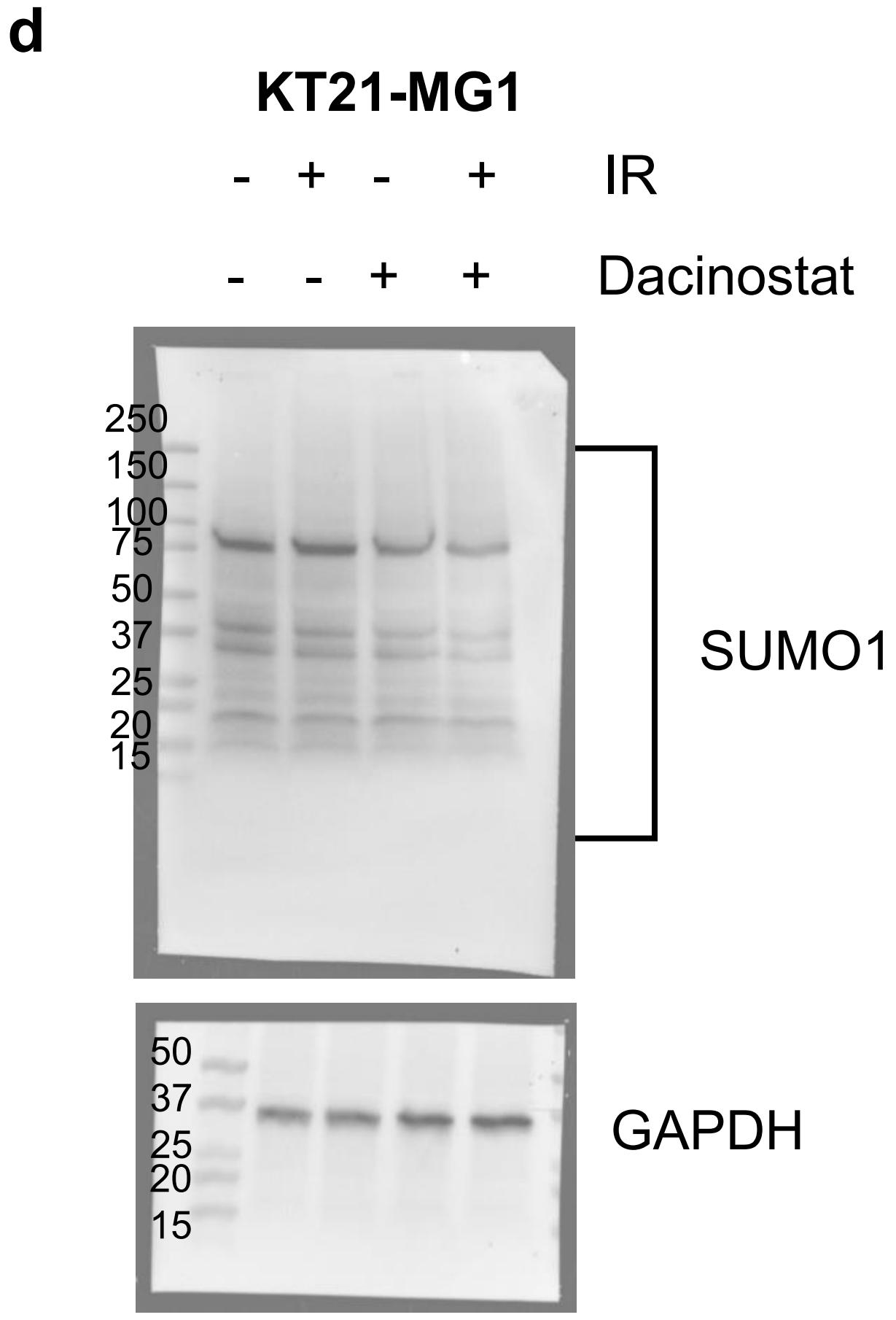

Supplement: Supplementary file 2 [file 41419_2026_8899_MOESM5_ESM.pdf]
